# Chromid-like secondary replicons as predicted key sites of biosynthetic gene clusters in *Ktedonobacteria*

**DOI:** 10.1128/msystems.00197-26

**Published:** 2026-05-29

**Authors:** Shuhei Yabe, Yu Zheng, Shunji Takahashi, Chongyang Yang, Yui Nose, Shinichi Yamazaki, Nao Okuma, Mazytha Kinanti Rachmania, Fitria Ningsih, Wellyzar Sjamsuridzal, Mayuko Sato, Kiminori Toyooka, Yasunori Ichihashi

**Affiliations:** 1 RIKEN Center for Sustainable Resource Science 98319, Tsukuba, Japan; 2 RIKEN Center for Sustainable Resource Science 98319, Wako, Saitama, Japan; 3 Department of Biology, Faculty of Mathematics and Natural Sciences, Universitas Indonesia 64733, Depok, Indonesia; 4 Center of Excellence for Indigenous Biological Resources-Genome Studies, Faculty of Mathematics and Natural Sciences, Universitas Indonesia 64733, Depok, Indonesia; 5 RIKEN Center for Sustainable Resource Science 98319, Yokohama, Japan; BRIC National Agri-Food and Biomanufacturing Institute, Mohali, Punjab, India

**Keywords:** *Ktedonobacteria*, biosynthetic gene clusters, chromid, secondary metabolism, comparative genomics, volcanic soils

## Abstract

Soils harbor immense biosynthetic gene cluster (BGC) diversity that mediates microbial interactions, yet this potential remains unevenly mapped and poorly characterized across diverse bacterial lineages. *Ktedonobacteria* (phylum *Chloroflexota*) are an actinomycete-like lineage widely distributed in terrestrial soils, including oligotrophic volcanic deposits; however, their secondary metabolism and genome architecture remain poorly characterized. Here, we integrate targeted cultivation from volcanic soils at Mount Zao (Japan) with genome-resolved metagenomics and comparative analysis of public genomes to examine biosynthetic potential across 183 ktedonobacterial genomes. We identified 1,546 BGCs and grouped them into 1,162 non-redundantgene-cluster families (GCFs) using antiSMASH and BiG-SLiCE. Nearly one quarter of genomes encoded≥10 distinct GCFs, and several family-level clades exhibited high GCF richness that approached that of *Streptomyces* within our data set, highlighting a putatively biosynthetically rich yet underexplored soil bacterial lineage. Most ktedonobacterial BGCs were highly divergent from current reference collections and exhibited unusually low intra-genomic redundancy, suggesting broad putative chemical diversity. Long-read assemblies from 10 cultured strains revealed recurrent 1.6–3.5 Mb ECE-like contigs with chromid-like features, but distinct maintenance features. These replicons were consistently enriched in BGCs and mobility-associated genes, with mobility loci concentrated near BGC boundaries. Collectively, our results expand the phylogenetic landscape of soil biosynthetic diversity and highlight ECE-like contigs as major genomic reservoirs for secondary metabolism in *Ktedonobacteria*.

**IMPORTANCE:**

Soil bacteria produce many of the small molecules that become medicines and help microbes interact with each other. Yet most of this chemical diversity remains unexplored because many soil lineages are difficult to cultivate and remain genomically underrepresented. Much of what we know comes from well-studied groups such as actinomycetes, leaving many soil lineages largely unexplored. We analyzed 183 genomes from *Ktedonobacteria*, an actinomycete-like group within the phylum Chloroflexota that is widespread in terrestrial soils, including nutrient-poor volcanic deposits. We uncovered a large and diverse set of gene clusters predicted to produce secondary metabolites, many of which lack close counterparts in current reference collections. We also show that these clusters are concentrated on large ECE-like contigs with chromid-like features, pointing to a dedicated genomic reservoir that can accumulate and reshuffle biosynthetic traits. Our results expand the known sources of soil biosynthetic diversity and provide a foundation for future cultivation and functional characterization of *Ktedonobacteria* metabolites.

## INTRODUCTION

Microbial secondary metabolism underpins diverse ecological interactions, including antagonism, resource acquisition, signaling, and stress tolerance (1). Soils harbor large reservoirs of biosynthetic gene clusters (BGCs) encoding chemically diverse metabolites, many of which are exploited as antibiotics, agrochemicals, and other bioactive compounds (2, 3). Yet, the breadth of lineages with high biosynthetic potential and the genomic architectures supporting this potential remain incompletely characterized (4). Extreme terrestrial habitats provide an informative setting because intense, fluctuating stresses may favor specialized metabolites that mediate niche construction and competition (5–8).

Within these extreme habitats, *Ktedonobacteria* (phylum *Chloroflexota*) are often abundant in pioneer microbial communities, especially in volcanic bare-ground soils where they likely contribute to early primary succession on oligotrophic substrates (9–13). These environments impose strong resource limitations and are characterized by fluctuating redox and climatic stresses, likely favoring flexible metabolic and defensive repertoires. *Ktedonobacteria* also exhibit a morphology akin to that of *Actinomycetes*, including filamentous growth, spores, and sporangia (14, 15), suggesting a potential coupling between complex life cycles and secondary metabolism.

Despite these features, the biosynthetic and ecological capacities of *Ktedonobacteria* remain underexplored compared with those of canonical discovery lineages such as *Streptomyces*. Individual strains have yielded unusual metabolites (15). For example, *Thermosporothrix hazakensis* produces rare acyloins and indole–thiazole derivatives (16). One of these compounds was previously optimized into ITE CONHCH₃, a synthetic aryl hydrocarbon receptor agonist effective in a murine colitis model (17). However, such examples provide only a fragmentary view of the chemical potential of *Ktedonobacteria*, and little is known about how secondary metabolites contribute to their ecology *in situ*. Cultured representatives remain scarce, and isolate genomes have increased only modestly in recent years (18), whereas hundreds of metagenome-assembled genomes (MAGs) for *Ktedonobacteria* are now available. This shift expands phylogenetic coverage, but MAG fragmentation and incomplete recovery of secondary replicons can obscure genome architecture and secondary metabolism (19, 20).

One dimension of genome architecture relevant to secondary metabolism is the presence of large extrachromosomal elements (ECEs), which can concentrate secondary metabolism-related genes and facilitate the horizontal transfer and diversification of biosynthetic repertoires (21–23). In *Streptomyces* and other bacterial genera, large ECEs (often classified as megaplasmids or chromids) serve as repositories of BGCs and underpin lineage-specific chemistry (23). Chromids combine plasmid-like replication/maintenance with chromosome (CHR)-like features (e.g., similar GC content/codon usage and carriage of core genes) and have been proposed to facilitate niche adaptation (22, 24). Several ECEs occur across multiple bacterial phyla (22, 25); however, their prevalence and functional roles within *Ktedonobacteria* remain poorly defined. In a previous study, we noted ECE-like contigs in several *Dictyobacteraceae* strains, but their signatures and biosynthetic contributions remained unresolved (15).

In the present study, we combine targeted cultivation of volcanic bare-ground soil samples from Mount Zao (Japan) with genome-resolved metagenomics and public genome analysis to assess secondary metabolism and replicon architecture across *Ktedonobacteria*. Our findings showed that BGC diversity is widespread across this class, that ECE-like contigs with chromid-like features recur across multiple families and are enriched in BGCs and mobility-related genes, and that biosynthetic repertoires vary with lineage and habitat.

## MATERIALS AND METHODS

### Soil sampling and TOC quantification

Samples of volcanic barren soil were collected on 29 September 2023 from the crater rim of Lake Okama, Mount Zao, Miyagi Prefecture, Japan (38.1321°N, 140.4468°E; 1,680 m above sea level; Fig. S1A). Fifteen surface soil samples (0–10 cm) were collected using sterile tools and transported on ice to the laboratory within 48 h. Based on vegetation cover and total organic carbon (TOC) content, the samples were classified as non-vegetated soil (NVS; *n* = 10, 0.25%–0.41% TOC), poorly vegetated soil (PVS; *n* = 3, 0.46%–0.99% TOC), and richly vegetated soil (RVS; *n* = 2, 2.04% TOC). Subsamples were stored at –20°C for DNA extraction and at 4°C or –80°C for cultivation. For TOC analysis, approximately 50 g of each sample was air-dried at 25°C. TOC was quantified by treating 0.5 g of soil with 2 M HCl to remove inorganic carbon, followed by 12 h of venting and freeze-drying. The carbon content was then measured using a multi EA 4000 analyzer (Analytikjena, Jena, Germany).

### DNA extraction, sequencing, and assembly and MAG processing

Selected soil samples were subjected to amplicon (16S rRNA gene) sequencing and shotgun metagenomic sequencing, and reads were processed using MetaWrap pipelines (26). Metagenomes were assembled and binned to recover MAGs, which were quality-filtered based on completeness and contamination thresholds and taxonomically assigned using GTDB-Tk (27). Detailed protocols, software versions, and parameters are provided in Supplementalmethods.

### Isolation, genome sequencing, and scanning electron microscopic (SEM) analysis of *Ktedonobacteria*

The isolation, identification, complete genome sequencing, and SEM analysis of *Ktedonobacteria* are described in Supplemental methods. Briefly, isolates were obtained by imprinting fresh soil onto 1/10-strength R2A medium solidified with 1.5% (wt/vol) gellan gum and 0.2% (wt/vol) CaCl₂·2H₂O. The medium was acidified to pH 5.0 with HCl and supplemented with 30 mg L⁻¹ sodium azide to suppress fast-growing heterotrophs. Plates were incubated aerobically at 30°C in the dark for 60 days. Putative *Ktedonobacteria* colonies, characterized by tough substrate-rooted mycelia, were identified by near full-length 16S rRNA gene sequencing (primers 27F/1492R) and the EZBioCloud bioinformatics platform (28). For SEM analysis, colonies were fixed overnight with 2% osmium tetroxide vapor at room temperature. Samples were then coated with osmium using an HPC-1SW coater and observed using a Hitachi SU8220 field-emission SEM at an accelerating voltage of 3 kV.

Two strains were fully assembled from long reads (Flye) (29) and polished/circularized using the Pilon (30) and Circlator (31) tools.

### Phylogenomics of *Ktedonobacteria*

The procedures for phylogenomic analysis are described in detail in Supplemental methods. Briefly, a data set comprising 184 ktedonobacterial genomes was compiled by combining public genomes from the National Center for Biotechnology Information with 21 MAGs derived from Mount Zao samples and two new isolates (Z7_2 and Z4_9). Public MAGs were dereplicated at 95%average nucleotide identity (ANI) using dRep (fastANI) (32). All genomes were assigned bacterial marker genes using GTDB-Tk v2.1.1 (bac120) (27). To maximize phylogenetic resolution, marker genes were aligned with gtdbtk align using the --skip_trimming option, rather than the default GTDB-Tk masking procedure that reduces the concatenated alignment to approximately 5,000 amino acid positions. This yielded a full-length concatenated aminoacid alignment of 35,833 sites, which was then trimmed with TrimAl v1.4 (33) (-gt 0.5 -cons 60) prior to maximumlikelihood tree inference in IQ-TREE v2.3.6 under the LG + F + G4 model with 1,000 ultrafast bootstrap replicates. Trees were visualized in iTOL (34). Family-level clades were delineated with TreeCluster (“max_clade”) using a distance threshold of 0.45 (35), and clade assignments were subsequently integrated with BGC profiles and replicon architecture in downstream comparative analyses. A summary of the genome collection, filtering, and downstream analysis workflow is provided in Fig. S2.

### BGC prediction, clustering, diversity, and distance-based novelty analysis

BGC prediction and comparative analyses are described in detail in Supplemental methods. Briefly, the analysis included a total of 183 ktedonobacterial genomes (excluding Merged_NVS_bin65), and BGCs were predicted using antiSMASH v7.1.0 (36), restricting the input to scaffolds ≥5kb to limit fragmentation bias (37, 38). BGCs were clustered into gene cluster families (GCFs) using BiG-SLiCE v2.0 at a cosine of 0.4 (39), and GCF counts were mapped onto the phylogeny (iTOL) (34). Following the metric recommended by Gavriilidou et al. (40), lineages were defined as BGC-rich when genomes harbored≥10 distinct GCFs, and clades were defined as highly BGC-rich when the mean per-genome GCF count across member genomes was≥15. GCFs were assigned to major biosynthetic classes based on antiSMASH/BiG-SLiCE annotations. Comparisons across lineages and genome types were tested using Wilcoxon rank-sum tests with Benjamini–Hochberg correction. Distance-based novelty was evaluated using (i) BiG-SLiCE v1.1.1 against the BiG-FAM data set (41), (ii) BiG-SCAPE against the MiBIG v4.0 database (42), and (iii) antiSMASH KnownClusterBlast (36). These metrics quantify divergence from reference BGCs in feature or similarity space and do not, by themselves, demonstrate confirmed chemical novelty.

### Identification and comparative analysis of ECE-like contigs

Candidate ECE-like contigs were first screened operationally from high-contiguity long-read assemblies (≤10 contigs) as contigs ≥1Mb whose normalized CheckM completeness was markedly lower than expected from contig length. The 1 Mb threshold was chosen based on previous work showing that chromids are generally larger than megaplasmids (mean 1.52 Mb vs 0.77 Mb; median 1.26 Mb vs 0.56 Mb) (22), whereas reduced normalized CheckM completeness was used because large secondary replicons are expected to encode few universal single-copy marker genes and therefore show disproportionately low CheckM completeness relative to their size. To distinguish large ECEs from fragmented CHRs, we compared observed CheckM completeness (domain Bacteria) to the expected value proportional to contig length (contig length/total genome size) and designated contigs as ECE-like when the normalized completeness was≤0.2, an operational threshold intended to capture large secondary replicons with disproportionately low marker-gene content relative to their size. These candidate contigs were then further characterized using multiple chromid-associated features, including replication-related signatures, GC content, and codon-usage similarity to the host CHR-like contig, and functional differentiation between replicons (Supplemental methods).

Replication-related features (e.g., strand asymmetry, predicted origins, and replication/partition genes) were profiled, and ECE-like contigs were compared with their cognate CHR-like contigs using genome similarity, orthology-based comparisons, and functional annotation (Supplemental methods). To test whether mobility-associated genes were depleted within BGCs and/or enriched near BGC boundaries, we quantified overlap and proximity between antiSMASH-predicted BGC intervals and mobility-gene intervals and evaluated enrichment using length-corrected tests, complemented by within-replicon permutations (Supplemental methods). Contig-scale synteny and feature tracks were visualized using Circos (43).

### Binning performance evaluation using a mock*Ktedonobacteria* metagenome

To benchmark recovery of ECE-like contigs, we constructed a mock metagenome from 10 *Ktedonobacteria* genomes, each comprising a primary CHR-like contig and one representative ECE-like contig. Multi-contig assemblies were merged into single continuous sequences, and the resulting replicon sequences were combined into a reference FASTA. Community heterogeneity was simulated by assigning genome abundances from a log-normal distribution (mean = −2, σ = 1.0), with identical abundances enforced for each CHR-like contig–ECE-like contig pair. Based on the sequencing output of our environmental metagenomic samples (40–44 million read pairs per sample), we simulated a comparable scale by generating 46 million paired-end Illumina reads (151 bp) using InSilicoSeq v1.5.4 (44). Reads were quality-controlled with MetaWRAP v1.3.2 (26) and assembled with MEGAHIT v1.2.9 (45). The assembly was binned with MaxBin2 v2.2.7 (46), MetaBAT2 v2.15 (47), CONCOCT v1.1.0 (48), and COMEBin (release 2023-10) (49) using MetaWRAP or native pipelines under default settings. Reconstructed bins were evaluated with CheckM v1.1.6 (50) and metaQUAST v5.2 (51) to estimate completeness, contamination, and genome fraction relative to the known references. Bins were then classified by origin as CHR (≥90% of a single chromosomal sequence), ECE (≥90% of a single ECE-like contig), Host_CHR_ECE (a CHR together with its cognate ECE), or Chimeric_CHR/Chimeric_CHR_ECE (mixtures from unrelated CHRs and/or ECEs). This scheme enabled a controlled comparison of binning performance for ECE recovery.

## RESULTS

### Distribution and recovery of *Ktedonobacteria* from Zao volcano soils

The Okama crater rim at Mount Zao is characterized by oligotrophic volcanic bare-ground soils interspersed with localized vegetated patches, providing a natural gradient of early-stage soil development (Fig. S1A). To investigate the distribution of *Ktedonobacteria*, we conducted 16S rRNA gene (V4 region) amplicon sequencing on 15 soil samples categorized by vegetation cover into NVS, PVS, and RVS (Data S1). *Ktedonobacteria* represented one of the dominant bacterial classes across samples (Fig. S1B). Notably, their relative abundance tended to decrease with increasing vegetation cover, with ranges of 3.0%–17.6%, 0.60%–12.22%, and 0.03%–4.94% in NVS, PVS, and RVS, respectively. This pattern was consistent with the preference for bare-ground soils observed in this data set, although the small sizes of PVS and RVS samples preclude statistical inference (Fig. S1C).

Metagenomic sequencing of five NVS and three PVS samples yielded a total of 166 MAGs (≥50% completeness,<10% contamination) spanning 12 bacterial phyla (52). dRep clustering (≥95%ANI) identified 90 species-level clusters, including 21 ktedonobacterial MAGs grouped into nine species-level clusters (Data S2, S3) (53).

We also attempted cultivation of *Ktedonobacteria* from the same samples. The colonies were screened on modified R2A medium under oligotrophic conditions, using pH adjustment and antibiotics to suppress fast-growing heterotrophs. Colonies with substrate-rooted mycelia were selected and identified via 16S rRNA gene sequencing. Among 56 colonies examined, four were assigned to *Ktedonobacteria*. After three rounds of purification, four axenic strains representing two species were obtained, designated as strains Z7_2 (JCM 37,047; DSM 118,755) and Z4_9 (JCM 37,048; DSM 118,756), respectively (Data S4).

### Phylogenetic structure of *Ktedonobacteria* based on MAGs and cultured genomes

**Fig 1:**
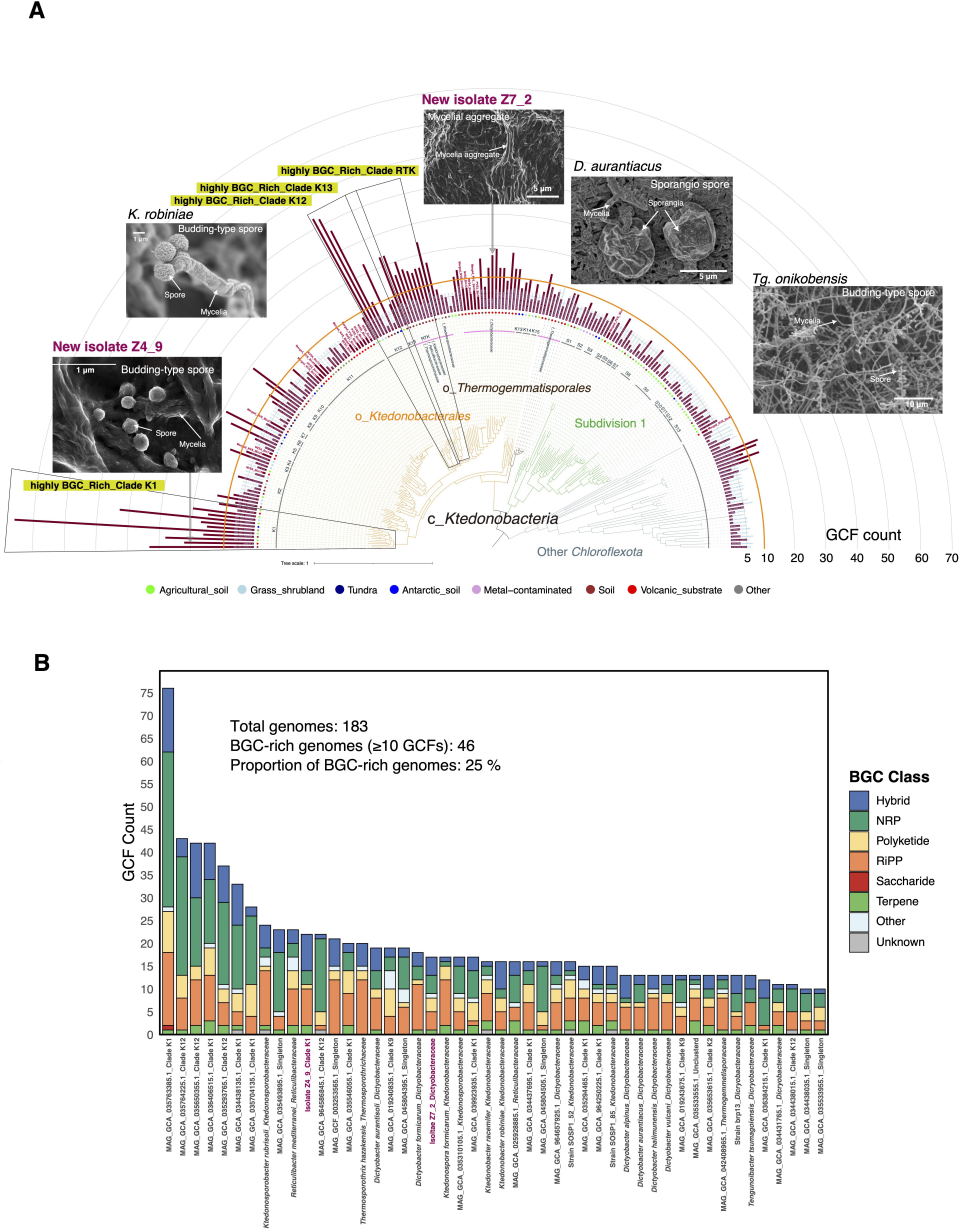
Phylogenetic structure, habitat distribution, and predicted biosynthetic capacity of *Ktedonobacteria*. (**A**) Maximum likelihood phylogeny inferred from the GTDB bac120 marker set (35,833 aligned amino acid positions) for 183 genomes (metagenome-assembled genomes, previously cultivated representatives, and the newly isolated strains Z7_2 and Z4_9 from Mount Zao samples). Order-level clades are color-coded. Radial bars denote the number of GCFs per genome (antiSMASH v7.1.0 calls clustered with BiG-SLiCE 2.0 at cosine 0.40). Point symbols indicate habitat categories as shown in the legend. Family-level clades within Ktedonobacterales (e.g., K1, K12, K13, and RTK) were delineated using TreeCluster (v1.0.4, max_clade mode, threshold 0.45). BGC-rich lineages were defined as genomes with ≥ 10 GCFs, and highly BGC-rich clades as family-level groups with ≥ 15 GCFs per genome. Representative scanning electron micrographs illustrate that actinomycete-like filamentous morphology is broadly conserved across distinct clades (scale bars as indicated). (**B**) Distribution of biosynthetic gene cluster classes across the 183 ktedonobacterial genomes examined. Bars show per-genome GCF counts, stacked by antiSMASH class and ordered by total GCFs. The inset summarizes the total number of genomes, the number of BGC-rich lineages (≥10 GCFs per genome), and their proportion. GCF, gene-cluster family; BGC, biosynthetic gene cluster.

A maximumlikelihood phylogeny was constructed using the GTDB bac120 marker set (35,833 aligned amino acid positions) (Fig. 1A). The data set included 21 MAGs from Mount Zao samples, 21 genomes of cultivated strains, the newly isolated strains Z7_2 and Z4_9, and 140 representative MAGs selected from 296 public ktedonobacterial MAGs (as of May 2025). Public MAGs were dereplicated at≥95%ANI to retain one representative per species-level cluster (Data S5).

The obtained phylogeny resolved three major lineages—*Ktedonobacterales*, *Thermogemmatisporales*, and an uncultivated “Subdivision 1”(Fig. 1A). TreeCluster (v1.0.4; max_clade; threshold = 0.45) was applied to define 37 operational phylogenetic clusters for downstream analyses (Data S5), denoted as Clade K (*Ktedonobacterales*) and Clade S (Subdivision 1) in Fig. 1A. Under this parameterization, *Ktedonobacteraceae*, *Reticulibacteraceae*, and *Thermosporotrichaceae* were merged as Clade RTK (formal family names retained in taxonomic contexts). Habitat information, antiSMASH-predicted BGC counts, and representative SEM images were mapped onto the tree (Fig. 1A). Twenty out of the 21 MAGs from Mount Zao samples corresponded to defined ktedonobacterial lineages, including Clades K2, K4, and K11, two novel singleton lineages, and the known *Dictyobacteraceae* family. One MAG (Merged_NVS_bin65) fell outside the core *Ktedonobacteria* clade (Fig. 1A), forming a distinct branch basal to other *Chloroflexota* taxa, and it was therefore excluded from downstream BGC analyses.

The newly isolated strain Z7_2 was grouped with *Dictyobacter arantiisoli* Uno17 but shared only 95.43% 16S rRNA gene identity (vs the 98.65% species threshold) (54), supporting its recognition as a previously undescribed species within *Dictyobacteraceae*. In contrast, Z4_9 fell within the uncultivated Clade K and showed 90.25% 16S rRNA identity to *D. arantiisoli*, consistent with a deeply branching lineage at approximately family-level divergence (Data S4).

### Predicted biosynthetic potential of *Ktedonobacteria* compared with *Streptomyces*

The secondary metabolic potential of 183 ktedonobacterial genomes (22 cultured, 161 MAGs) was assessed using antiSMASH v7.1.0, resulting in the identification of 1,546 BGCs, which were then dereplicated using BiG-SLiCE 2.0 into 1,162 non-redundant GCFs at a cosine threshold of 0.40. This cosine-based metric was adopted following Gavriilidou et al. (40), who recommended it because it reduces domain-count bias and better reflects BGC similarity than raw Euclidean distance (Supplementalmethods; Data S6). Using the same workflow for other *Chloroflexota* classes, *Streptomyces*, and representative *Actinomycetota*, we identified 46 species-level lineages (25%) as “BGC-rich” (≥10 GCFs per genome) (Fig. 1B). Within *Ktedonobacterales*, four TreeCluster clades (K1, K12, K13, and RTK) were highly BGC-rich (≥15 GCFs per genome), and the newly isolated strain Z4_9 was identified as belonging to the BGC-rich Clade K1.

**Fig 2:**
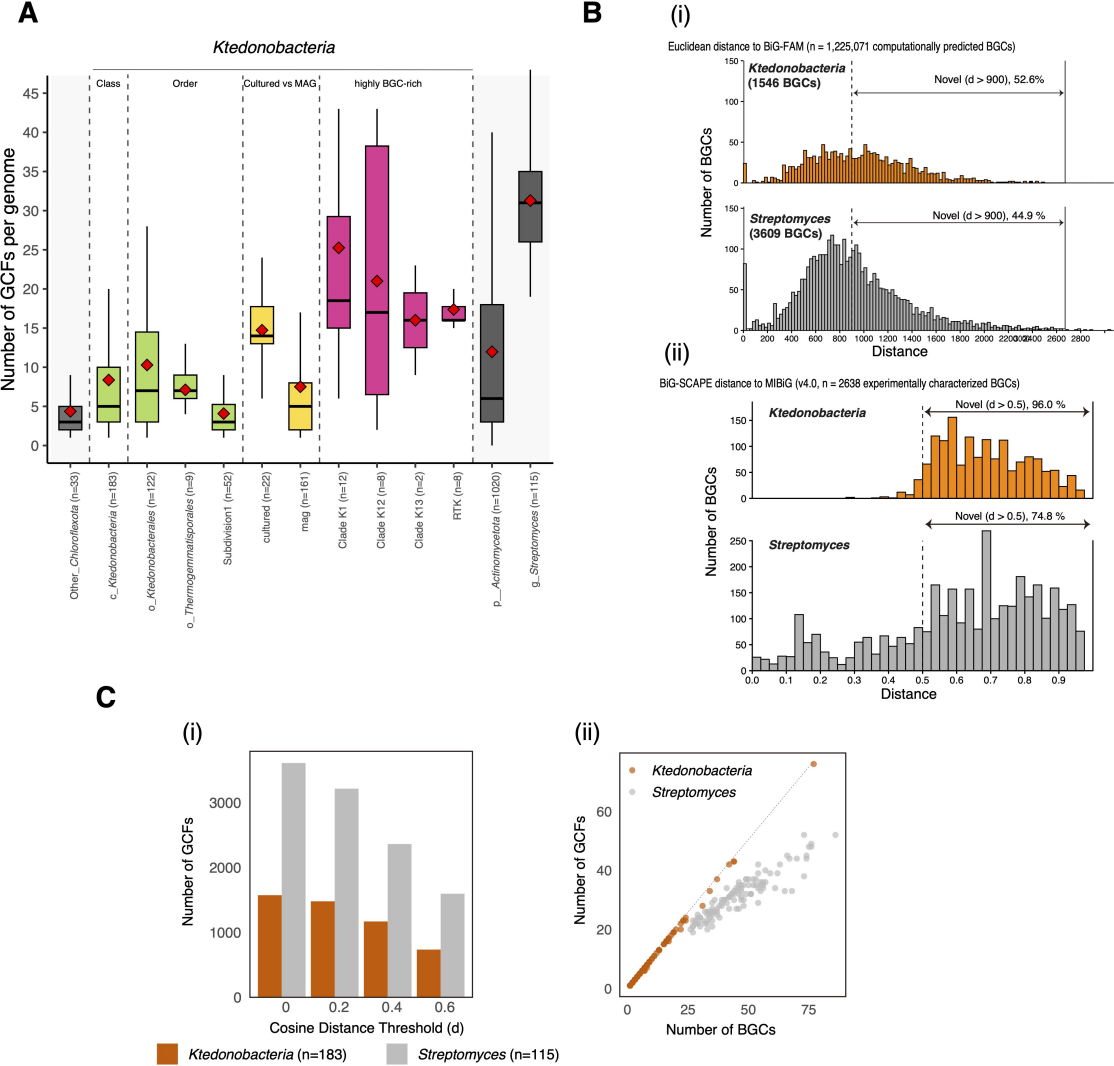
Comparative analysis of BGC abundance, distance-based putative novelty, and redundancy in *Ktedonobacteria*versus*Streptomyces*. (**A**) Number of GCFs per genome across groups. Box-and-whisker plots summarize per-genome GCF counts for orders within *Ktedonobacteria* (including cultured strains and MAGs), other classes of *Chloroflexota*, genus *Streptomyces*, and broader *Actinomycetota*. Boxes represent the IQR with the median line; whiskers extend to 1.5× IQR. Red diamonds indicate the mean value for each group. GCFs were obtained by running antiSMASH v7.1.0 and clustering the resulting BGCs with BiG-SLiCE 2.0 at a cosine of 0.40. Statistical comparisons were performed using Wilcoxon rank-sum tests with BH correction. *Ktedonobacteria*vs other Chloroflexota, Hodges–Lehmannshift = 2, 95%CI = 1–4, *P* = 0.006; cultured *Ktedonobacteria*vs MAGs, Hodges–Lehmannshift = 9.0, 95%CI = 6.0-11.0, *P* = 3.2 × 10⁻⁷; cultured *Ktedonobacteria*vs Actinomycetota, Hodges–Lehmannshift = 6.0, 95%CI = 2.0–9.0, *P* = 0.011. Full results are provided in Data S8. (**B**) Distance-based novelty of BGCs. (i)Distribution of Euclidean distances to the BiG-FAM database data set for the BGCs of *Ktedonobacteria* and *Streptomyces* (1,225,071 BGCs); larger distances indicate lower similarity to known families. (ii) Distribution of BiG-SCAPE distances (**D**)from each BGC to its closest MIBiG BGC; a higher *d* value indicates lower similarity. Proportions above the indicated thresholds are annotated in the panels. These distance-based metrics indicate divergence from current reference BGC data sets and should not be interpreted as direct evidence of confirmed chemical novelty. (**C**) Redundancy and threshold sensitivity. (i)Sensitivity of GCF counts to the BiG-SLiCE cosine threshold (results summarized across a range of thresholds). (ii) Relationship between the numbers of BGCs and GCFs per genome for *Ktedonobacteria* and *Streptomyces*, illustrating that ktedonobacterial genomes tend toward a near one-to-one correspondence (low redundancy), whereas *Streptomyces*genomes more oftenharbormultiple BGCs within the same GCF. GCF, gene-cluster family; BGC, biosynthetic gene cluster; IQR, interquartile range.

Across genomes, *Ktedonobacteria* averaged 8.4 GCFs per genome (*n* = 183), nearly twice the number observed for other *Chloroflexota* classes (4.4; *n* = 38; Hodges–Lehmann location shift = 2, 95%CI = 1–4, *P* = 0.006) (Fig. 2A). Cultured *Ktedonobacteria* were particularly BGC-rich (14.7; *n* = 22), exceeding ktedonobacterial MAGs (7.5; *n* = 161; Hodges–Lehmann location shift = 9.0, 95%CI = 6.0-11.0, *P* = 3.2 × 10⁻⁷) (Fig. 2A) and actinomycetotal genomes (12.0; *n* = 1,020; Hodges–Lehmann location shift = 6.0, 95%CI = 2.0–9.0, *P* = 0.011). This marked difference between cultured genomes and MAGs is consistent with incomplete recovery of large BGCs and/or secondary replicons in fragmented MAG assemblies. To further assess this possibility, we performed a mock metagenome binning benchmark using complete CHR-like and ECE-like contigs from 10 representative *Ktedonobacteria* genomes. Across four widely used binning tools, ECE-like contigs were recovered inconsistently and were frequently merged with cognate CHR-like contigs or incorporated into chimeric bins, indicating that current metagenomic binning workflows can underrecover or misassign large secondary replicons (Data S7). Notably, the most BGC-rich ktedonobacterial genomes reached GCF counts within the *Streptomyces* range (Fig. 2A). Within the *Ktedonobacteria* class, *Ktedonobacterales* and *Thermogemmatisporales* showed higher GCF counts than Subdivision 1 (Data S8).

Distance-based comparisons against current BGC reference data sets indicated that ktedonobacterial BGCs diverged substantially from current references (Fig. 2B), with 52.6% showing low similarity (Euclidean distance>900) against the BiG-FAM data set, compared with 44.9% of BGCs showing low similarity in *Streptomyces*[(Fig. 2B(i)]. When using BiG-SCAPE against the MIBiG database, 96.0% of ktedonobacterial BGCs exceeded *d* = 0.5 (vs 74.8% for *Streptomyces*) [Fig. 2B(ii)]. Consistently, KnownClusterBlast reported<20% similarity for>80% of ktedonobacterial BGCs, with only 5% (80/1,546) showing≥80% similarity to known clusters (Data S9).

Sensitivity analysis across BiG-SLiCE cosine thresholds from 0.0 to 0.6 showed that, although absolute GCF counts varied with threshold stringency, *Ktedonobacteria* and *Streptomyces* were affected in broadly similar ways [Fig. 2C(i)]. However, the former showed a ratio of approximately 1:1 between BGC and GCF counts per genome, consistent with low intra-genomic redundancy, whereas the latter more often carried multiple BGCs within the same GCF [Fig. 2C(ii)].

Collectively, these results indicate that some ktedonobacterial clades have predicted biosynthetic capacities comparable to those of *Streptomyces* and that many BGCs are highly divergent from current references. Because database-based comparisons are limited by incomplete and biased coverage, chemical characterisation will be required to confirm novelty and function.

### Lineage- and habitat-associated structuring of biosynthetic repertoires

Uniform manifold approximation and projection of subclass-level BGC profiles separated genomes primarily by phylogenetic lineage and, to a lesser extent, by habitat (Fig. S3; Data S10). Habitat labels, which were coarse categories derived from BioSample metadata, were interpreted as broad ecological descriptors rather than precise environmental classifications, and category sizes were uneven. PERMANOVA confirmed significant clustering by lineage (pseudo-F = 11.2, *R*²=0.71, *P* = 0.001) and by habitat (pseudo-F = 2.42, *R*²=0.09, *P* = 0.011), indicating that evolutionary history explained most of the variance in biosynthetic profiles, whereas habitat accounted for a comparatively small fraction.

**Fig 3:**
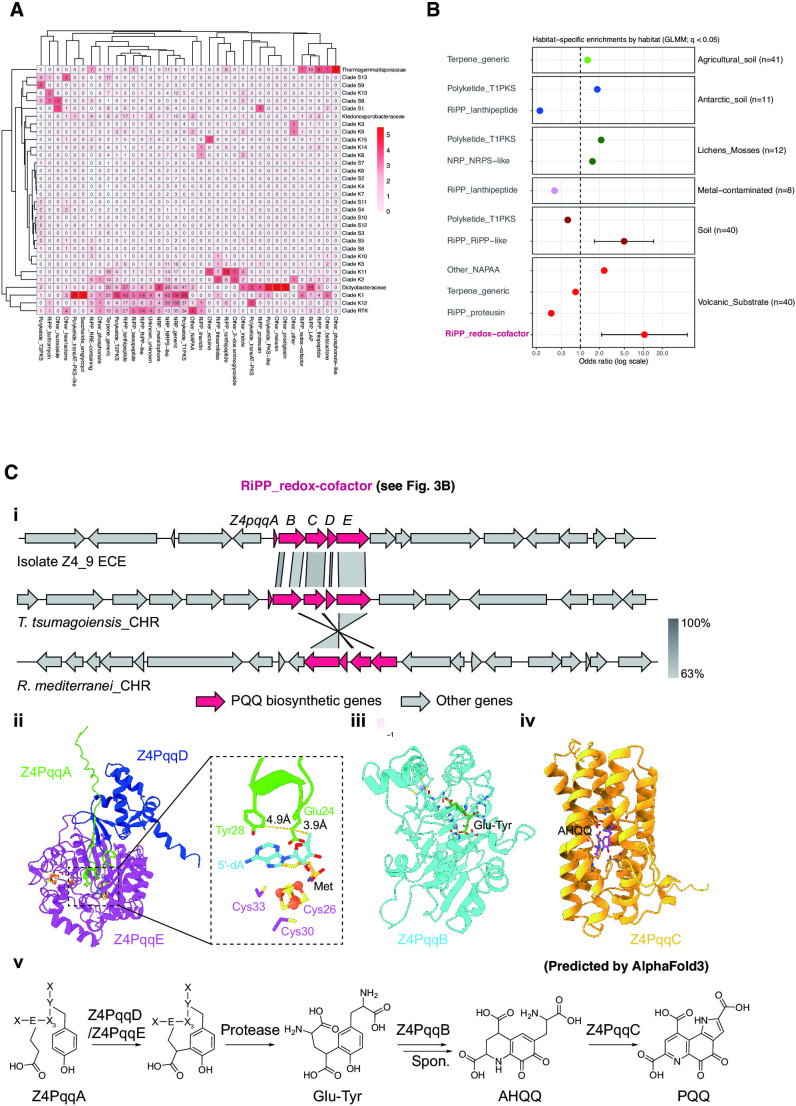
Phylogenetic structuring of ktedonobacterial biosynthetic repertoires and a putative RiPP_redox-cofactor pathway. (**A**) Heatmap showing the distribution of biosynthetic categories across taxonomic groups. Each row represents a taxonomic group and each column a biosynthetic category assigned at the GCF level (shown on the *x*-axis as “Class_Subclass”; e.g., RiPP_bottromycin). Genomes assigned to “Singleton” were excluded. To emphasize category-specific enrichment across taxa, values were *Z*-score-normalized per column, and original (untransformed) counts are overlaid as numbers in each cell. GCFs were classified using BiG-SLiCE, and biosynthetic categories were assigned based on antiSMASH/BiG-SLiCE annotations as described in Materials and Methods. For hybrid-type GCFs, all associated biosynthetic categories were counted independently.(**B**) Habitat-associated subclass signals inferred using phylogenetically informed logistic regression. Points indicate odds ratios for BGC subclass–habitat category combinations, and error bars indicate 95% confidence intervals. Habitat categories were based on broad BioSample-derived metadata and should be interpreted cautiously. No individual subclass–habitat association remained significant after Benjamini–Hochberg correction; therefore, these patterns represent nominal habitat-associated signals rather than statistically robust habitat-specific enrichment. (**C**) RiPP_redox-cofactor locus and PQQ-like locus inference. (i)Comparative analysis of RiPP_redox-cofactor clusters annotated by antiSMASH from the ECE of strain Z4_9, CHR of *Thermogemmatispora tsumagoiensis*, and CHR of *Reticulibacter mediterranei*. Genes involved in PQQ biosynthesis (pqqA–E) are shown in red and other genes in gray; sequence identity is indicated by gray shading. (ii) AlphaFold 3-predicted complex model of Z4PqqA, Z4PqqD, and Z4PqqE with the SAM-derived products 5′-dA and Met. The Glu24 and Tyr28 residues of the EXXXY motif in Z4PqqA are shown as sticks, and distances are indicated by yellow dashed lines. (iii) and (iv) AlphaFold 3-predicted complex models of Z4PqqB bound to the Glu–Tyr substrate (panel iii) and Z4PqqC bound to the AHQQ intermediate (panel iv). Key residues surrounding the ligands are shown as sticks. (v)Putative functional model for Z4PqqA–E in strain Z4_9 within a PQQ-like (peptide-derived redox-cofactor) locus, inferred from gene content and structure predictions. The proposed maturation scheme is based on previously established models (55).

Lineage-level comparisons revealed distinct biosynthetic signatures (Fig. 3A). *Thermogemmatisporaceae* were enriched in RiPP subclasses (including thiopeptides and RRE-containing peptides) and beta-lactones, whereas Clade K11 exhibited a predominance of lantipeptides and terpenes and lacked polyketide synthase (PKS)-related clusters. *Dictyobacteraceae* uniquely encoded traits such as melanin/prodigiosin biosynthesis, whereas BGC-rich clades (K1, K12, and RTK) were enriched in PKS and non-ribosomal peptide synthetase clusters.

To address phylogenetic non-independence more explicitly, we re-analysed habitat associations of BGC subclasses using phylogenetically informed logistic regression. Although several subclass–habitat combinations showed nominal signals before correction, no individual association remained significant after Benjamini–Hochberg correction. The strongest uncorrected signal involved RiPP_redox-cofactor clusters in genomes assigned to volcanic-substrate categories (odds ratio = 4.55, 95%CI = 1.64–12.65, *P* = 0.0037, *q* = 0.169; 14/40 focal genomes versus 11/141 non-focal genomes) (Data S11). We therefore interpret habitat-related patterns as suggestive rather than conclusive, particularly given the coarse habitat labels, uneven category sizes, and the possibility that residual sampling structure remains difficult to disentangle from shared ancestry.

Consistent with this cautious interpretation, RiPP_redox-cofactor clusters were detected in genomes from Mount Zao samples, including Z4_9, as well as in additional genomes assigned to both volcanic and non-volcanic habitat categories. These clusters showed low similarity to known references in KnownClusterBlast (13%–20% similarity to lankacidin C) and contained conserved pqqA–E-like genes consistent with PQQ-like loci (Data S9). Thus, the observed distribution is compatible with occurrence in volcanic-associated genomes but does not support a statistically robust habitat-specific enrichment once phylogeny and multiple testing are taken into account.

To further characterize RiPP_redox-cofactor clusters, we examined Z4_9 together with the type strains *Tengunoibacter tsumagoiensis* and *Reticulibacter mediterranei*. All three genomes were shown to harbor highly syntenic loci [Fig. 3C(i)] and, in Z4_9, the locus encodes pqqA–E in the canonical order. AlphaFold3 predictions were consistent with a canonical PQQ biosynthetic pathway, including a RiPP chaperone-like PqqD, a radical SAM-like PqqE, and PqqB/PqqC folds compatible with downstream oxidative steps [Fig. 3C(ii–v)]. No pqqF homolog was identified within these loci, suggesting that the proteolysis required for maturation may be provided in *trans* (55). Accordingly, a genome-encoded protease that complements the function of PqqF might be involved in PQQ production.

### Large ECE-like contigs with chromid-like features in *Ktedonobacteria*

To link BGCs with large ECEs, 20 ktedonobacterial genomes (18 type strains plus Z4_9 and Z7_2) were scanned for large contigs distinct from the primary CHR. For the initial screening, ECE-like contigs were defined operationally as≥1Mbp sequences whose CheckM-estimated completeness was≥5-fold lower than expected based on their size relative to the total genome, which restricted the analysis to high-contiguity long-read assemblies (≤10 contigs) (Table 1; Data S12; cf. chromid and megaplasmid size distributions in diCenzoandFinan [22]). Using this definition, 10 genomes (eight type strains and both new isolates) with at least one such replicon were identified; the remainder had a single CHR, lacked≥1Mbp contigs, or were too fragmented to be evaluated.

**TABLE 1:** Summary of chromosome- and ECE-like contigs, genome features, and marker gene distribution in newly isolated and representative *Ktedonobacteria* genomes^*b*^

Taxon	Putative topology	TIR length	Genome size (bp) [# contigs]	Completeness (%)	Marker gene (GTDB bac120)	16S rRNA copies	GC content (%)	Mean coverage
Family *Dictyobacteraceae*								
Isolate Z7_2 (BAAJPE000000000)	NA	NA	8,900,022 [2]	98.3	117	6	49.59	NA
Chromosome-like contig	Unclear	ND	5,378,691	98.3	115	5	49.36	495.8
ECE-like contig	Linear	4.3 kb	3,521,331	1.7	2	1	49.93	493.8
*Dictyobacter aurantiacus* S27^T^ (GCF_003967515.1)	NA	NA	8,881,661 [2]	100.0	117	9	53.97	NA
Chromosome-like contig	Linear	10.9 kb	6,133,338	98.3	110	7	54.39	NA
ECE-like contig	Linear	10.9 kb	2,748,323	3.3	7	2	53.05	NA
*Dictyobacter kobayashii* Uno11^T^ (GCF_003967555.1)	NA	NA	8,854,844 [2]	100.0	115	9	50.28	NA
Chromosome-like contig	Unclear	ND	6,031,971	98.3	109	7	50.35	NA
ECE-like contig	Unclear	ND	2,822,873	2.4	6	2	50.13	NA
*Dictyobacter halimunensis* S3.2.2.5^T^ (GCF_036245075.1)	NA	NA	9,414,265 [2]	98.3	116	9	54.27	NA
Chromosome-like contig	Unclear	ND	5,914,761	98.3	110	7	54.71	NA
ECE-like contig	Unclear	ND	3,423,416	2.7	6	2	53.55	NA
*Dictyobacter alpinus* Uno16^T^ (GCF_003967575.1)	NA	NA	8,961,382 [4]	100.0	117	9	49.70	NA
Chromosome-like contig	Linear	9.4 kb	5,582,978	98.3	112	7	49.8	NA
ECE-like contig	Linear	5.1 kb	3,135,874	2.0	5	2	49.25	NA
*Dictyobacter vulcani* W12^T^ (GCF_008974265.1)	NA	NA	7,209,578 [7]	98.2	112	9	49.44	NA
Chromosome-like contig	Unclear	ND	4,245,503	94.7	109	7	49.22	NA
ECE-like contig	Unclear	ND	1,572,654	0.0	3	2	49.89	NA
*Tengunoibacter tsumagoiensis* Uno3^T^ (GCF_003967535.1)	NA	NA	7,699,857 [2]	98.3	117	9	49.36	NA
Chromosome-like contig	Circular^*a*^	ND	5,303,167	98.3	115	8	49.46	NA
Circular ECE-like contig	Circular^*a*^	ND	2,396,690	0.6	2	1	49.14	NA
Family *Ktedonobacteraceae*								
*Ktedonobacter robiniae* SOSP1-30^T^ (GCF_016587375.1)	NA	NA	10,717,923 [9]	100.0	115	8	51.63	NA
Chromosome-like contig	Unclear	ND	4,907,184	96.6	113	7	54.02	NA
ECE-like contig	Unclear	ND	2,645,227	0.9	2	1	53.31	NA
Family *Reticulibacteraceae*								
*Reticulibacter mediterranei* 150040^T^ (GCF_016587455.1)	NA	NA	12,668,978 [4]	100.0	117	5	51.98	NA
Chromosome-like contig	Unclear	ND	8,906,807	99.1	116	5	51.81	NA
ECE-like contig	Unclear	ND	3,085,112	0.9	1	0	52.45	NA
Undescribed family								
Isolate Z4_9 (BAAJPD010000000)	NA	NA	9,913,309 [2]	100.0	117	4	50.38	NA
Chromosome-like contig	Unclear	ND	7,616,622	98.3	115	4	50.39	570.6
ECE-like contig	Unclear	ND	2,296,687	0.0	2	0	50.35	637.4

^*a*^ The circular topology of *Tengunoibacter tsumagoiensis* Uno3T was reported previously (15).

^*b*^ This table summarizes the presence of putative extrachromosomal element (ECE)-like contigs in two newly isolated strains from Mt. Zao and selected genomes of the class *Ktedonobacteria*. ECE-like contigs were defined as ≥1 Mbp contigs whose CheckM-based completeness was at least five times lower than the completeness expected from their size relative to the total genome. CheckM-based completeness was assessed for each contig of the selected genomes. “Putative topology” indicates whether the contig was inferred to be circular, linear, or unclear, based on terminal inverted repeats (TIRs) or assembly structure; ND,not determined, NA,not applicable. TIR length refers to the approximate size of terminal inverted repeats, where detected. Genome sizes are shown together with the total number of contigs in parentheses (see Data S12 for details of small contigs). Mean Illumina coverage was calculated only for the two newly isolated strains Z7_2 and Z4_9.

**Fig 4:**
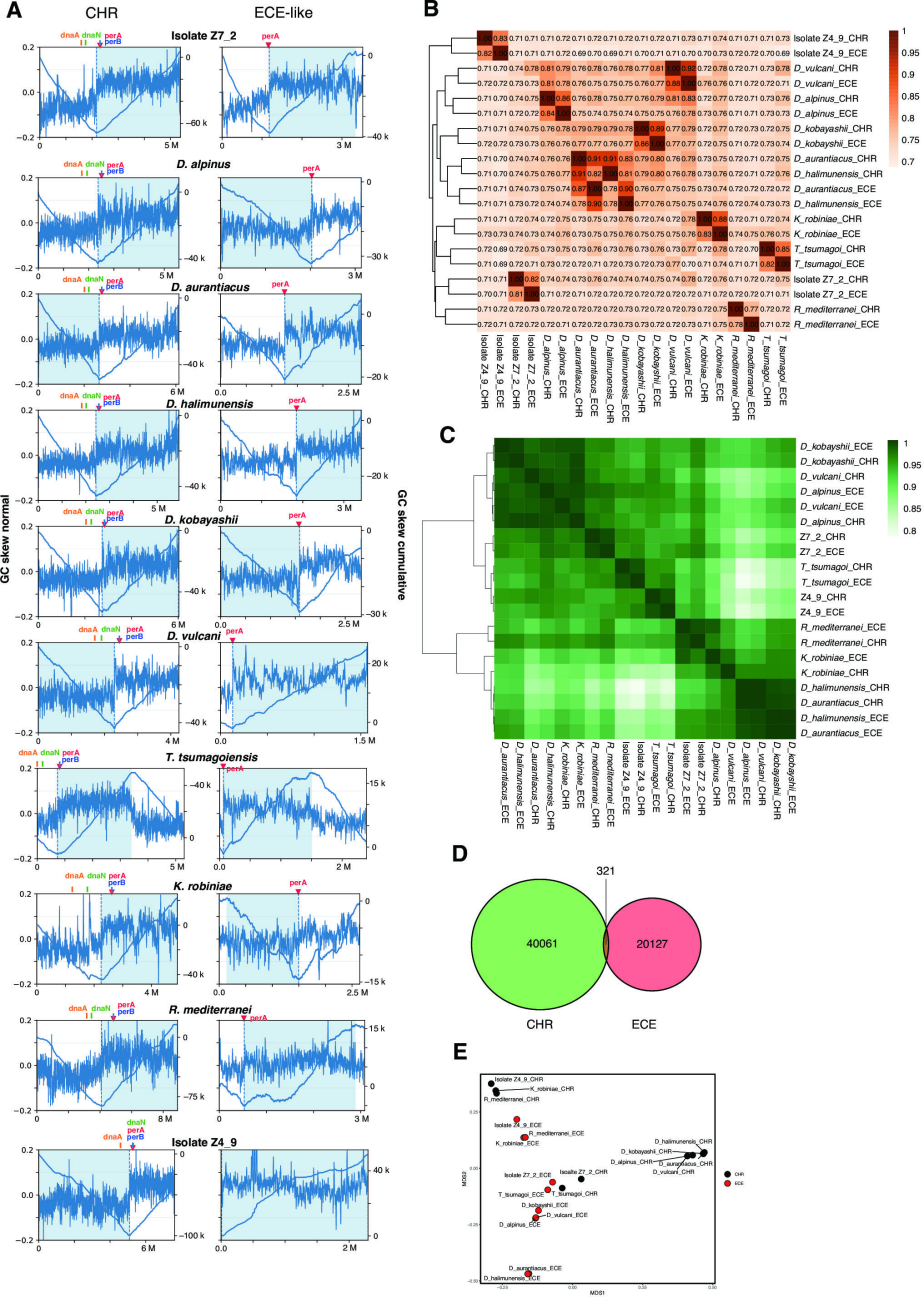
Genomic and compositional distinctions between CHR- and ECE-like contigs in the class *Ktedonobacteria*. (**A**) Cumulative GC skew profiles of CHR- and ECE-like contigs. Paired plots of normalized GC skew (blue line, left *y*-axis) and cumulative GC skew (shaded curve, right *y*-axis) are shown for each representative CHR–ECE pair. Gene markers for *dnaA*, *dnaN*, *parA*, and *parB* are indicated above each plot. The ori, predicted using Ori-Finder 2022, is shown by vertical dashed lines. The *x*-axis represents genomic coordinates (Mb), and the right *y*-axis for cumulative GC skew is scaled in arbitrary units (K).(**B**) Heatmap of ANI among CHR- and ECE-like contigs, calculated using PyANI (ANIb). Values indicate pairwise ANI and are shown by color intensity (and numbers). Host–ECE pairs are highlighted with black circles. (**C**) Heatmap of codon-usage correlation (Spearman’s rank) among CHR- and ECE-like contigs. (**D**) Venn diagram showing the number of orthologous genes shared between CHR- and ECE-like contigs and those detected exclusively in each group. (**E**) Comparison of CHR- and ECE-like contigs based on gene-content variation. PCoA was performed for gene presence/absence patterns across 7,586 orthologous gene clusters (identified using Panaroo in strict mode). Pairwise dissimilarities were calculated using Jaccard distance. Each point represents a CHR- or ECE-like contig; filled and open symbols indicate CHR- and ECE-like replicons, respectively. PERMANOVA confirmed a statistically significant separation between the two groups (pseudo-F = 2.04, *R*²=0.10, *P* = 0.004). ori, origin of replication; ANI, average nucleotide identity; PCoA, principal coordinate analysis; ECE, extrachromosomal element; CHR, chromosome.

Most ECE-like contigs encoded only 1–7 GTDB bac120 marker genes and often harbored 16S rRNA genes. Cumulative GC-skew (Z-curve) profiles showed distinct V- or inverted V-shaped extrema on CHR- and ECE-like contigs, consistent with putative replication-origin (ori) candidates in all strains except Z4_9 (Fig. 4A); the major V coincided with the ori predicted by Ori-Finder 2022. In CHR-like contigs, dnaA/dnaN (replication initiation) were colocalized with parA/parB (partitioning) as a contiguous locus. In contrast, Pfam HMM searches detected no canonical Rep-family initiators on ECE-like contigs; parA homologs were present on all ECE-like contigs except those from Z4_9 and, when present, localized near the putative ori peak, whereas parB was not detected on any ECE-like contig (Fig. 4A). Consistent with distinct maintenance systems, the obtained ParA/RepA phylogeny separated CHR- and ECE-derived homologs into two well-supported groupings; most ECE-like contig ParA sequences formed a coherent clade, whereas ParA from Z7_2 and *Ktedonobacter robiniae* branched independently (Fig. S4).

Although the GC content of the ECE-like contig was nearly identical to that of the host CHR in each strain (Table 1), the replicons were nucleotide-dissimilar (fastANI 77–92%[Fig. 4B]; Mash 0.22–0.26 based on 15-mers [Data S13; Fig. 4B]). Despite this divergence, ECE-like codon usage correlated most strongly with the host CHR, and the two replicons often clustered together (Fig. 4C). In *D. halimunensis* and *D. aurantiacus*, the CHR-like and ECE-like contigs of each strain formed a pair, consistent with ANI-based clustering. For Z7_2 and Z4_9, mean Illumina coverage was~1:1 for CHR- and ECE-like contigs (Table 1).

Pan-genome analysis in Panaroo identified 40,061 genes unique to CHR-like contigs, 20,127 genes unique to ECE-like contigs, and only 321 shared genes (Fig. 4D), indicating strong compositional partitioning. Ortholog sharing among ECE-like contigs was sparse and largely strain-specific (Data S14). Jaccard-based PCoA separated CHR- and ECE-like contigs into distinct clusters (Fig. 4E), which was supported by PERMANOVA results (pseudo-F = 2.04, *R*²=0.10, *P* = 0.004).

Functionally, CHR-like contigs were enriched in housekeeping processes, whereas ECE-like contigs were enriched in transport/metabolism, secondary metabolism, and mobile functions (COG P, G, Q, K, and L) (Fig. S5). Taken together, these ECE-like contigs share key chromid-like properties (e.g., large size, GC content, and sequencing coverage similar to the primary CHR, host-correlated codon usage, and ParA-based maintenance signatures), which distinguish them from ordinary plasmids.

### BGC enrichment and associations with mobility genes in ECE-like contigs with cross-replicon homology

**Fig 5:**
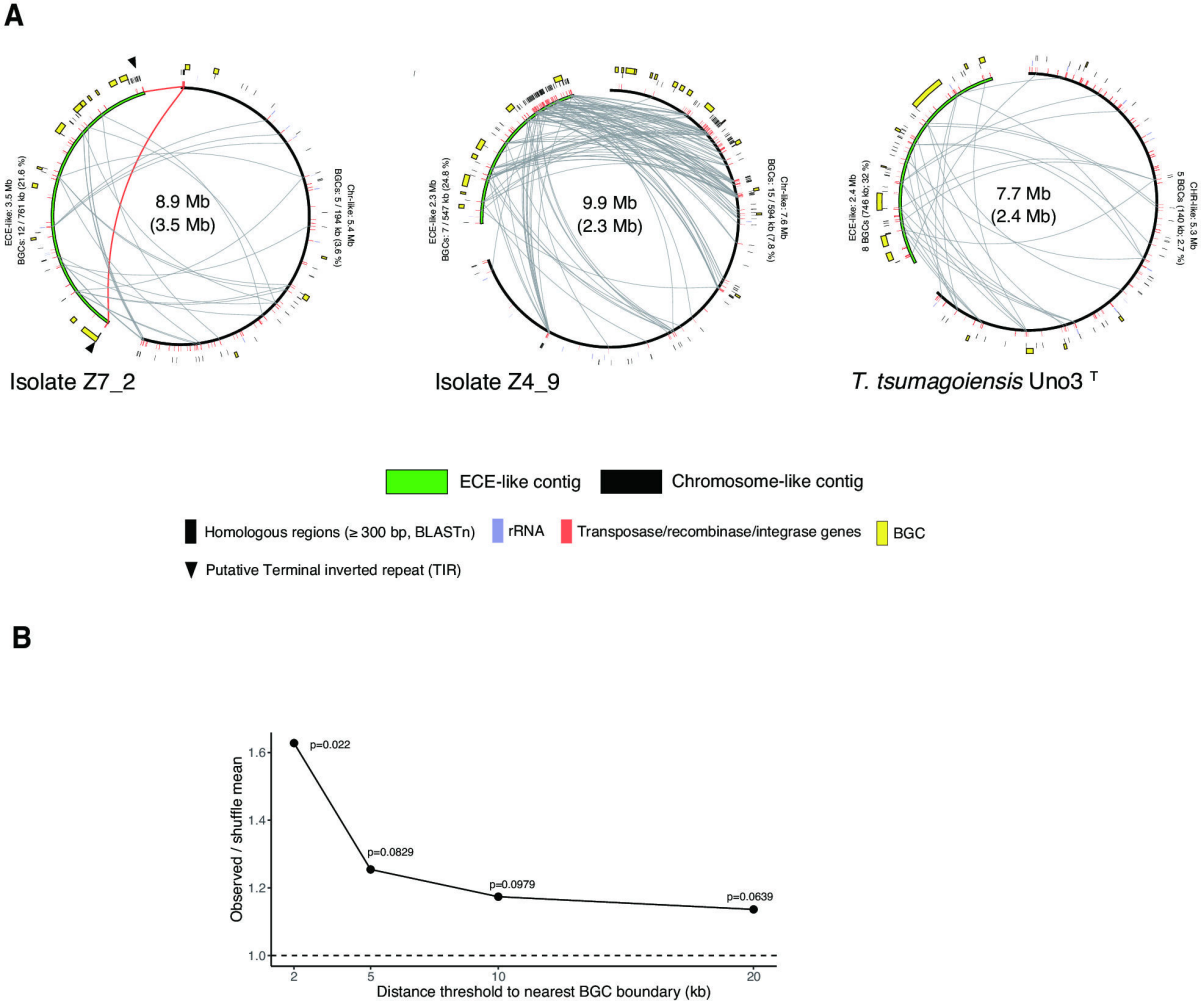
Representative structural organization of CHR- and ECE-like contigs and the spatial association of mobility loci with BGC boundaries. (**A**) Circular maps showing representative CHR–ECE pairs from Isolate Z7_2, Isolate Z4_9, and *Tengunoibacter tsumagoiensis* Uno3. CHR- and ECE-like contigs are shown in black and green, respectively. Gray ribbons connect pairwise homologous regions detected by BLASTn (identity ≥ 98%, length ≥ 300bp). Orange ribbons indicate long, high-identity homologous segments (length ≥ 5kb, identity ≥ 99%). Black triangles denote putative terminal inverted repeats (TIRs). Gene features are overlaid as follows: yellow boxes, biosynthetic gene clusters (BGCs) predicted with antiSMASH v7.1.0; red ticks, mobility-associated genes (integrases, recombinases, and transposases); purple ticks, rRNA operons. The center of each panel shows the total length of the CHR–ECE pair, with the ECE-like contig length in parentheses. Ring-side labels report replicon length and BGC burden in the format “8 BGCs (746 kb; 32%),” where the percentage indicates the fraction of the corresponding replicon covered by BGCs. (**B**) Cumulative enrichment of mobility loci located within increasing distance thresholds from the nearest BGC boundary, shown as the ratio of the observed count to the mean count obtained by contig-restricted shuffling (1000 permutations). Mobility loci overlapping BGC bodies were excluded from this analysis. The dashed horizontal line indicates the null expectation under the shuffle-based model (observed/shuffle mean = 1). Empirical one-sided permutation *P* values are shown for each distance threshold. Together, these results indicate that mobility loci are preferentially concentrated in the immediate vicinity of BGC boundaries, with the strongest enrichment detected within approximately 2 kb. TIR, terminal inverted repeat; ECE, extrachromosomal element; BGC, biosynthetic gene cluster; CHR, chromosome.

The genome maps of 10 strains were annotated for BGCs, mobility-associated genes (integrases, recombinases, and transposases), and high-identity segments shared between CHR- and ECE-like contigs (≥98% nucleotide identity over≥300bp) (Fig. 5; Fig. S6). Each genome encoded 25–238 predicted mobility-associated genes distributed across both replicons, and in several cases, the shared high-identity segments overlapped these loci (Fig. 5;Fig. S6). Putative terminal inverted repeats (TIRs;≥5kb,≥99% identity), often considered hallmarks of linear replicons, were detected on CHR- and ECE-like contigs in *D. aurantiacus* and *D. alpinus*, and only on the ECE-like contigs in strain Z7_2. Consistent with this topology, cross-replicon homology blocks (≥5kb and≥99% identity) were mapped to the respective termini in all three strains, a pattern consistent with recent or ongoing recombination or segmental exchange between CHR- and ECE-like contigs.

BGCs occurred on both replicons but were generally enriched on ECE-like contigs. The fraction of each ECE-like contig sequence carrying BGCs exceeded that of the corresponding CHR-like contig in all strains examined and was particularly large in *T. tsumagoiensis* (32.0%), *D. vulcani* (31.1%), Z4_9 (24.8%), and Z7_2 (21.6%) (Fig. 5;Fig. S6).

Length-corrected binomial tests and within-replicon shuffling were used to quantify spatial associations between mobility loci and BGCs (Fig. S7A through C). Across replicons, mobility-associated loci were depleted within BGC bodies (fold enrichment = 0.78, 95%CI = 0.65–0.93; binomial *P* = 0.0023), but enriched in the flanking regions of BGCs, with the strongest effect observed within 2 kb of BGC boundaries (fold enrichment = 1.77, 95%CI = 1.10–2.70; binomial *P* = 0.0099). Overall, these analyses indicate that mobility loci preferentially occurred near BGC boundaries rather than inside BGCs. Mobility-associated loci outside BGCs were also overrepresented near BGC boundaries, particularly within 2 kb, where the observed frequency was 1.63-fold higher than the contig-restricted shuffle expectation (95%interval = 1.05–3.50; permutation *P* = 0.022 (Fig. S7C;Data S15).

### Differences in BGC subclass composition between CHR- and ECE-like contigs

**Fig 6:**
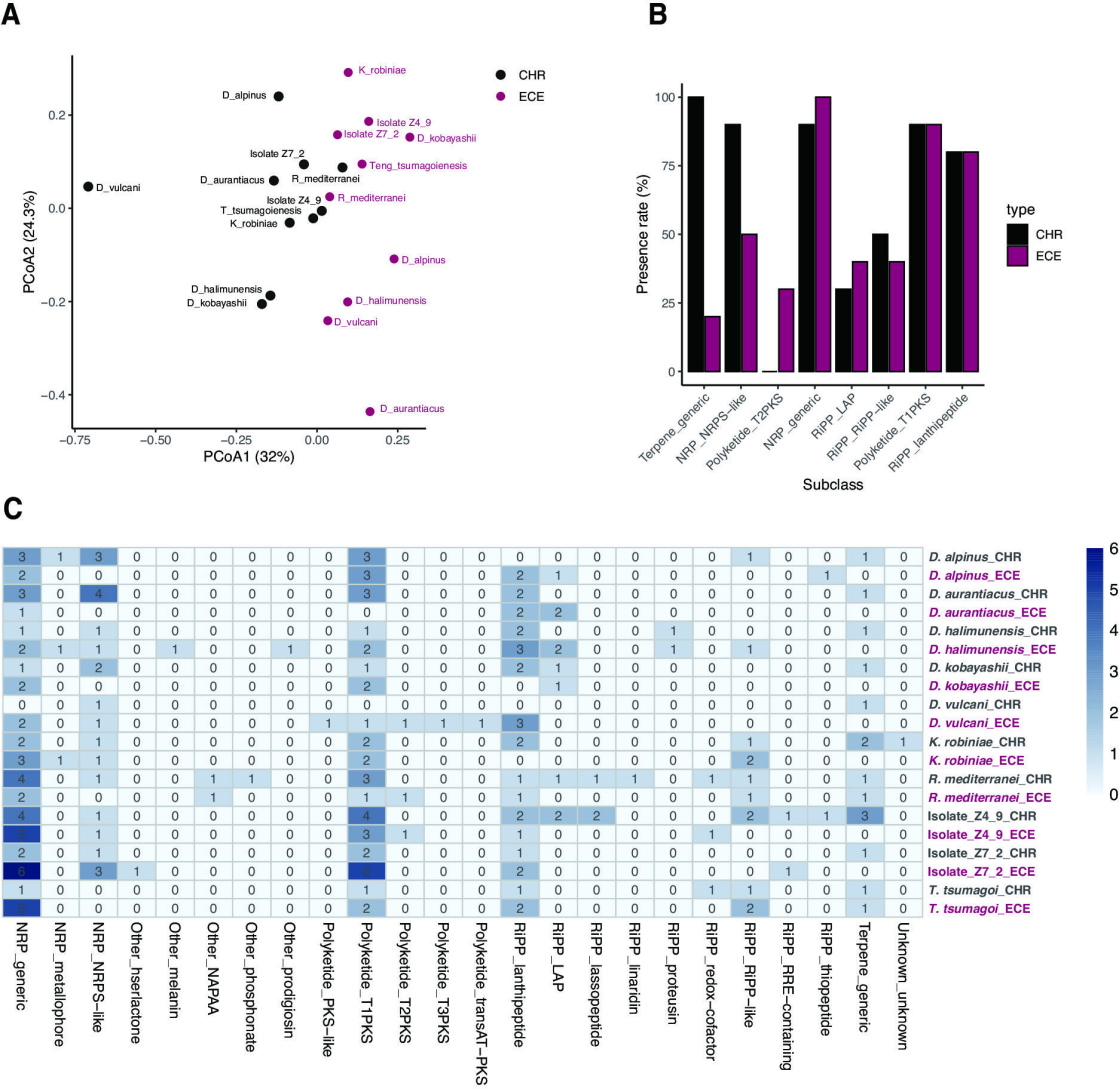
Subclass-level distribution of biosynthetic gene clusters on CHR- and ECE-like contigs in *Ktedonobacteria*. (**A**) PCoA of Bray–Curtis dissimilarities calculated from subclass-level BGC relative abundances. Points are differently colored based on replicon type (CHR-like contig, black; ECE-like contig, red) and labeled with genome identifiers. PERMANOVA results showed significant separation by replicon type (pseudo-F = 3.42, *R*²=0.16, *P* = 0.001). (**B**) Bar plot showing presence rates (%) of BGC subclasses in CHR- (black) and ECE-like contigs (red). Only subclasses detected in at least three genomes were included. No subclasses were significant after multiple-testing correction (exact McNemar test with Benjamini–Hochberg adjustment; *P* ≥ 0.05). (**C**) Heatmap showing the counts of GCFs assigned to each BGC subclass across replicons. Rows and columns correspond to individual replicons and BGC subclasses, respectively. Cell shading indicates the number of detected GCFs per subclass, with darker shades indicating higher counts. GCF, gene-cluster family; BGC, biosynthetic gene cluster; PCoA, principal coordinate analysis; ECE, extrachromosomal element; CHR, chromosome.

Principal coordinate analysis of subclass-level BGC profiles separated CHR- and ECE-like contigs (Fig. 6A), as confirmed by PERMANOVA results (pseudo-F = 3.42, *R*²=0.16, *P* = 0.001), indicating a replicon-type structuring of BGC repertoires.

Subclass frequency comparisons showed no significant differences after multiple-testing correction (Fig. 6B), but terpene clusters were largely confined to CHR-like contigs, whereas type II PKS clusters were detected only on ECE-like contigs in our data set.

Heatmap visualisation showed genome-level replicon signatures, with several BGC subclasses encoded exclusively on ECE-like contigs (Fig. 6C). For example, in Z7_2, a homoserinelactone (hserlactone) cluster was confined to the ECE-like contig, and in *D. halimunensis*, multiple subclasses (including RiPP-related and pigment-associated clusters) occurred only on ECE-like contigs.

### Assessment of binning performance for ECE-like contigs using a mock metagenome

Standard metagenome binning pipelines often apply genome completeness thresholds, typically>50%, based on universal single-copy marker genes (36, 56). Although this works well for recovering near-complete CHR-like contigs, it can exclude large ECE-like contigs, which often lack these markers because of their atypical gene content. On the other hand, if completeness-based filtering is not applied, large ECE-like contigs may still be recovered as separate bins because they are distinct from host CHR-like contigs in nucleotide identity and k-mer composition (Fig. 4B). However, their GC content (Table 1) and sequencing coverage can be similar to those of the host CHR-like contig, which may lead to merging or misbinning. We therefore tested how well current binning tools can separate large ECE-like contigs from CHR-like contigs under controlled conditions.

To do this, we constructed a mock metagenome using 10 representative *Ktedonobacteria* strains, each represented by one CHR-like contig and one ECE-like contig. To remove the effects of fragmentation, each replicon was treated as a single contig. Relative abundances were assigned using a log-normal distribution (μ = −2, σ = 1.0), with equal abundance for the CHR-like and ECE-like contigs from each strain. We then simulated 46 million paired-end Illumina reads (151 bp), approximating the sequencing depth of our Mount Zao metagenomic data sets. The reads were assembled with MEGAHIT and binned using MaxBin2, MetaBAT2, CONCOCT, and COMEBin.

Binning performance varied substantially among the tools (Data S7). MaxBin2 produced six bins, but only one, corresponding to the Z4_9 CHR, was correctly reconstructed; the others were chimeric and contained either unrelated CHR-like fragments or mixtures of CHR-like and ECE-like contigs. MetaBAT2 generated 21 bins, including nine correctly reconstructed CHR-like contigs and eight distinct ECE-like contigs, with four chimeric bins. CONCOCT produced six bins; in four of them, each ECE-like contig was merged with its corresponding CHR-like contig, and the remaining two bins were chimeric. COMEBin showed similar performance to MetaBAT2, recovering eight CHR-like contigs and six ECE-like contigs, with five chimeric bins. Overall, tools that relied mainly on sequence composition and coverage tended to recover ECE-like contigs more successfully, whereas methods that depended more strongly on single-copy marker genes more often failed to separate them correctly.

Together, these results show that commonly used binning tools have clear limitations for recovering large ECE-like contigs. Even under idealized conditions, using complete and unfragmented replicons with controlled abundance distributions, only a subset of ECE-like contigs were accurately recovered. This suggests that large ECE-like replicons are likely to be underrepresented, merged into chromosomal bins, or misclassified in many MAG-based studies, potentially obscuring their contributions to microbial adaptation and secondary metabolism.

## DISCUSSION

*Ktedonobacteria* is a morphologically distinctive lineage within the phylum *Chloroflexota* (15, 57) and is often abundant in extreme terrestrial environments, including volcanic soils and polar regions (Fig. 1A) (9, 10, 13, 58). Our analyses indicated that this bacterial class encodes a rich and compositionally novel repertoire of predicted BGCs, many of which are located on ECE-like contigs with chromid-like features. The isolated representative of the previously uncultivated, BGC-enriched Clade K1, together with the *Dictyobacter* isolate described here, provides a basis for experimental validation and future exploration of their secondary metabolites.

Overall, 25% of the 183 dereplicated ktedonobacterial genomes examined were shown to encode ≥10 non-redundant GCFs each (Fig. 1B), indicating that BGC richness is shared across multiple families (Fig. 1A) rather than restricted to a few taxa. This trait was observed across several family-level clades (e.g., K1, K12, K13, and RTK), consistent with phylogenetically widespread biosynthetic potential. Distance-based comparisons suggest that *Ktedonobacteria* represent a promising, but still bioinformatically inferred, source of biosynthetic diversity comparable in some respects to canonical discovery lineages: against the BiG-FAM data set (1.225 million predicted BGCs)(39), 52.6% of ktedonobacterial BGCs exceeded the novelty threshold (Euclidean distance >900) (41) versus 44.9% for *Streptomyces*[Fig. 2B(i)]; whereas against the MIBiG data set (59), 96.0% exceeded the novelty cutoff (*d* > 0.5) (60) [Fig. 2B(ii)]. Clustering analysis further indicated low intra-genomic redundancy, with an approximately one-to-one correspondence between BGCs and GCFs per genome, contrasting with the results obtained for *Streptomyces* (Fig. 2C). Overall, these patterns suggest that individual ktedonobacterial genomes encode compact, structurally diverse BGC repertoires, which may benefit discovery efforts that prioritize breadth over closely related chemotypes. An important caveat is that these estimates are likely conservative. Cultured genomes harbored roughly twice as many GCFs as MAGs on average (Fig. 2A), consistent with incomplete recovery of large BGCs and secondary replicons in fragmented metagenome-assembled genomes. This interpretation is further supported by our mock metagenome benchmark, in which ECE-like contigs were frequently merged with cognate CHR-like contigs or recovered as part of chimeric bins, depending on the binning algorithm (Data S7). Accordingly, estimates of BGC abundance and ECE-like contig prevalence derived from MAGs should be regarded as conservative.

A salient ecological question is why *Ktedonobacteria* carry such abundant and diverse BGCs. Secondary metabolism appears to be associated with morphological complexity (61): the K1 strain Z4_9 and the *Dictyobacter* strain Z7_2 harbor numerous distinct GCFs and exhibit complex filamentous morphologies (Fig. 1A and B). Strain Z4_9 belongs to a deeply branching, previously uncultivated clade that likely represents a novel, BGC-enriched family, whereas Z7_2 expands cultivation-based diversity within *Dictyobacteraceae*. Filamentous, actinomycete-like morphologies were widely observed across *Ktedonobacteria* (Fig. 1A). This pattern is consistent with a possible association between secondary metabolism and morphological differentiation, potentially relevant to surface-associated growth and interactions within microbial communities. However, this interpretation is currently supported only at the genomic level, and functional validation will be required to determine whether these BGCs are actually expressed or regulated in association with developmental stages. Although a relationship between these traits has been well documented in *Actinomycetes* (61), whether *Ktedonobacteria* use an analogous strategy remains to be tested. The isolates examined in this study provide an opportunity for direct tests linking genome architecture, morphology, ecological function, and secondary metabolites.

BGC distributions differed across phylogenetic ranks (family and order; Fig. 3A;Fig. S3), whereas only a weak association was detected between broad habitat categories and secondary-metabolite potential (Fig. S3; Data S11). This weaker pattern may largely reflect shared ancestry, consistent with the preferential vertical preservation of core biosynthetic pathways (62). In phylogenetically informed analyses, no individual habitat–subclass association remained significant after multiple-testing correction. RiPP_redox-cofactor clusters nevertheless represented the strongest nominal signal, being observed in several genomes assigned to broad, volcanic-associated habitat categories. However, given the coarse habitat annotation, uneven sampling, and lack of significance after correction, this pattern should be regarded as suggestive rather than as evidence for a robust ecological preference. This observation may be compatible with previous proposals linking PQQ-like systems to oxidative or nutrient-limited settings (55, 63), but the present data are insufficient to infer such an ecological role directly. Whether these loci are expressed and what products, if any, they yield, remains to be determined.

Another key question is why BGCs may preferentially accumulate on ECE-like contigs. Representative cultured strains from several ktedonobacterial families have been shown to harbor large ECE-like contigs (1.6–3.5 Mb) with properties distinct from those of typical small plasmids (22). In each strain examined in the present study, the GC content and sequencing coverage of the ECE-like contig were nearly identical to the primary CHR, and yet the former showed only 77%–92% ANI and substantial gene-content divergence (Fig. 4A, B, and D; Table 1). Codon-usage patterns of the two replicons were strongly correlated and often clustered together (Fig. 4C), and the obtained ParA/RepA phylogeny recovered distinct but coherent clades for CHR- versus ECE-derived homologs (Fig. S4). Functional profiles support this separation. CHR-like contigs were enriched in housekeeping processes, whereas ECE-like contigs were enriched in transport, secondary metabolism, and mobility functions (Fig. S5). Collectively, these observations indicate that these ECE-like contigs are large, host-adapted replicons with chromid-like features. They share nucleotide composition and translational signatures with the primary CHR (22, 24) and yet carry a distinct, functionally specialized gene repertoire (22). Although their evolutionary origin remains uncertain, these host-associated signatures and the enrichment in adaptive functions provide a plausible context for BGC accumulation on ECE-like replicons.

The ecological and evolutionary roles of chromids remain a subject of debate, but a widely discussed view is that they allow adaptive, environmentally responsive genes to accumulate on a secondary replicon while retaining core housekeeping functions on the primary CHR (22, 24, 64, 65). If the ECE-like contigs with chromid-like features in *Ktedonobacteria*fulfill a similar role, their large size and bias toward transport, secondary metabolism, and mobility functions would be consistent with such a division of labor and may contribute to the relatively large genome sizes observed in this bacterial class (15).

Our analyses indicate that mobility loci are depleted within BGCs but enriched near BGC boundaries, consistent with preferential insertion/rearrangement at cluster edges and potential reshuffling of biosynthetic modules while maintaining internal cluster integrity (66).

Extensive homologous segments are shared between CHR- and ECE-like contigs, and genes related to environmental adaptation are concentrated on ECE-like replicons (Fig. 5;Fig. S6). These patterns are consistent with mobile-element activity and homologous recombination facilitating the exchange of environmentally responsive genes, including BGCs, between replicons (56). One possible scenario is that ECE-like replicons undergo a comparatively rapid evolutionary change (22, 67), sampling and reshuffling adaptive loci, some of which may later become stabilized on the primary CHR, implying a functional division of labor between replicons.

Consistent with this interpretation, terpenes involved in carotenoid biosynthesis were broadly detected on CHR-like contigs (Fig. 6B), consistent with their conserved roles in light and oxidative stress responses (68). In contrast, ECE-like contigs frequently encoded BGC subclasses absent from the corresponding CHR (Fig. 6C). This pattern was particularly evident in *D. halimunensis*, whose ECE-like contig harbored RiPP_RiPP-like, prodigiosin, melanin, and metallophore clusters absent from the CHR (Fig. 6C). In environments subjected to fluctuations or disturbance, such ECE-restricted modules might provide flexible, niche-dependent functions that can be gained or lost over short evolutionary timescales (22).

Terminal homology between CHR- and ECE-like contigs was detected in a subset of ktedonobacterial genomes, consistent with occasional homologous recombination.

In summary, by integrating newly cultivated representatives with metagenome-derived genomes, we provide a genome-wide assessment of secondary metabolism and replicon architecture in *Ktedonobacteria*. Our analyses indicate clade-wide enrichment of diverse BGCs with low similarity to current reference data sets and reveal the pervasive presence of ECE-like contigs with chromid-like features that preferentially accumulate BGCs and exhibit mobility-associated functions. While these analyses reveal broad predicted biosynthetic diversity and substantial divergence from current reference data sets, the inferred novelty and functional roles of individual BGCs remain to be experimentally validated. These findings establish a foundation for future research to validate ECE autonomy and maintenance and to functionally characterizse ECE-borne BGCs through expanded cultivation and targeted experiments.

## Data Availability

Shotgun metagenome reads are available in the DDBJ Sequence Read Archive (DRA) under BioProject PRJDB35689 (runs DRR728410–DRR728417). Raw isolate genome reads (Z7_2 and Z4_9) are available under the same BioProject PRJDB35689 (runs DRR905084–DRR905085 and DRR905086–DRR905087). Metagenome-assembled genome (MAG) assemblies generated in this study have been deposited in DDBJ/ENA/GenBank (see Data S3 for the full list of GCA accession numbers). Complete genome assemblies of strains Z7_2 and Z4_9 have been submitted to DDBJ/ENA/GenBank under WGS accession numbers BAAJPE000000000 and BAAJPD010000000, respectively. The 16S rRNA gene sequences of these isolates are available under LC811933 (Z7_2) and LC811932 (Z4_9). Additional information is available from the corresponding author upon reasonable request.

## SUPPLEMENTAL MATERIAL

Supplemental Figures msystems.00197-26-s0001.pdf Figures S1-S7.

Supplemental Text msystems.00197-26-s0002.pdf Supplemental methods.

Supplemental Data msystems.00197-26-s0003.xlsx Data S1 to S15.

ASM does not own the copyrights to Supplemental Material that may be linked to, or accessed through, an article. The authors have granted ASM a non-exclusive, world-wide license to publish the Supplemental Material files. Please contact the corresponding author directly for reuse.

## References

[1] Dinglasan JLN, Otani H, Doering DT, Udwary D, Mouncey NJ. 2025. Microbial secondary metabolites: advancements to accelerate discovery towards application. Nat Rev Microbiol 23:338–354. doi:10.1038/s41579-024-01141-y 39824928

[2] Rutledge PJ, Challis GL. 2015. Discovery of microbial natural products by activation of silent biosynthetic gene clusters. Nat Rev Microbiol 13:509–523. doi:10.1038/nrmicro3496 26119570

[3] Crits-Christoph A, Diamond S, Butterfield CN, Thomas BC, Banfield JF. 2018. Novel soil bacteria possess diverse genes for secondary metabolite biosynthesis. Nature 558:440–444. doi:10.1038/s41586-018-0207-y 29899444

[4] Salamzade R, Kalan LR. 2025. Context matters: assessing the impacts of genomic background and ecology on microbial biosynthetic gene cluster evolution. mSystems 10:e01538-24. doi:10.1128/msystems.01538-24 39992097 PMC11915812

[5] Medeiros W, Hidalgo K, Leão T, de Carvalho LM, Ziemert N, Oliveira V. 2024. Unlocking the biosynthetic potential and taxonomy of the Antarctic microbiome along temporal and spatial gradients. Microbiol Spectr 12:e00244-24. doi:10.1128/spectrum.00244-24 38747631 PMC11237469

[6] Sayed AM, Hassan MHA, Alhadrami HA, Hassan HM, Goodfellow M, Rateb ME. 2020. Extreme environments: microbiology leading to specialized metabolites. J Appl Microbiol 128:630–657. doi:10.1111/jam.14386 31310419

[7] Andreani-Gerard CM, Cambiazo V, González M. 2024. Biosynthetic gene clusters from uncultivated soil bacteria of the Atacama Desert. mSphere 9:e00192-24. doi:10.1128/msphere.00192-24 39287428 PMC11520301

[8] Geers AU, Michoud G, Busi SB, Peter H, Kohler TJ, Ezzat L, Team T, Battin TJ, Styllas M, Schön M, Tolosano M, Staercke VD, Peter H, Kohler T, Battin TJ. 2025. Deciphering the biosynthetic landscape of biofilms in glacier-fed streams. mSystems 10:e01137-24. doi:10.1128/msystems.01137-24 39745394 PMC11834409

[9] Tebo BM, Davis RE, Anitori RP, Connell LB, Schiffman P, Staudigel H. 2015. Microbial communities in dark oligotrophic volcanic ice cave ecosystems of Mt. Erebus, Antarctica. Front Microbiol 6:179. doi:10.3389/fmicb.2015.00179 25814983 PMC4356161

[10] Hernández M, Calabi M, Conrad R, Dumont MG. 2020. Analysis of the microbial communities in soils of different ages following volcanic eruptions. Pedosphere 30:126–134. doi:10.1016/S1002-0160(19)60823-4

[11] Fantom N, Dawson RA, Prondvai E, Constant P, King GM, Schäfer H, Hernández M. 2025. Metabolism of CO and H2 by pioneer bacteria in volcanic soils and the phyllosphere. ISME J 19:wraf053. doi:10.1093/ismejo/wraf053 40089988 PMC12021596

[12] Jiang X, Takacs-Vesbach CD. 2017. Microbial community analysis of pH 4 thermal springs in Yellowstone National Park. Extremophiles 21:135–152. doi:10.1007/s00792-016-0889-8 27807621

[13] Yabe S, Sakai Y, Abe K, Yokota A. 2017. Diversity of Ktedonobacteria with actinomycetes-like morphology in terrestrial environments. Microbes Environ 32:61–70. doi:10.1264/jsme2.ME16144 28321007 PMC5371077

[14] Yabe S, Zheng Y, Wang C, Sakai Y, Abe K, Yokota A, Donadio S, Cavaletti L, Monciardini P. 2021. Reticulibacter mediterranei gen. nov., sp. nov., within the new family Reticulibacteraceae fam. nov., and Ktedonospora formicarum gen. nov., sp. nov., Ktedonobacter robiniae sp. nov., Dictyobacter formicarum sp. nov. and Dictyobacter arantiisoli sp. nov., belonging to the class Ktedonobacteria. Int J Syst Evol Microbiol 71. doi:10.1099/ijsem.0.004883 34296987

[15] Zheng Y, Saitou A, Wang C-M, Toyoda A, Minakuchi Y, Sekiguchi Y, Ueda K, Takano H, Sakai Y, Abe K, Yokota A, Yabe S. 2019. Genome features and secondary metabolites biosynthetic potential of the class Ktedonobacteria Front Microbiol 10:893. doi:10.3389/fmicb.2019.00893 31080444 PMC6497799

[16] Park J-S, Yabe S, Shin-ya K, Nishiyama M, Kuzuyama T. 2015. New 2-(1’H-indole-3’-carbonyl)-thiazoles derived from the thermophilic bacterium Thermosporothrix hazakensis SK20-1(T). J Antibiot (Tokyo) 68:60–62. doi:10.1038/ja.2014.93 25052483

[17] Grycová A, Joo H, Maier V, Illés P, Vyhlídalová B, Poulíková K, Sládeková L, Nádvorník P, Vrzal R, Zemánková L, Pečinková P, Poruba M, Zapletalová I, Večeřa R, Anzenbacher P, Ehrmann J, Ondra P, Jung J-W, Mani S, Dvořák Z. 2022. Targeting the aryl hydrocarbon receptor with microbial metabolite mimics alleviates experimental colitis in mice. J Med Chem 65:6859–6868. doi:10.1021/acs.jmedchem.2c00208 35416668

[18] Rachmania MK, Ningsih F, Sari D, Sakai Y, Yokota A, Yabe S, Kim S-G, Sjamsuridzal W. 2024. Dictyobacter halimunensis sp. nov., a new member of the phylum Chloroflexota, from forest soil in a geothermal area. Int J Syst Evol Microbiol 74:006600. doi:10.1099/ijsem.0.006600 39630498 PMC12453558

[19] Kerkvliet JJ, Bossers A, Kers JG, Meneses R, Willems R, Schürch AC. 2024. Metagenomic assembly is the main bottleneck in the identification of mobile genetic elements. PeerJ 12:e16695. doi:10.7717/peerj.16695 38188174 PMC10771768

[20] Maguire F, Jia B, Gray KL, Lau WYV, Beiko RG, Brinkman FSL. 2020. Metagenome-assembled genome binning methods with short reads disproportionately fail for plasmids and genomic Islands. Microb Genom 6:e000436. doi:10.1099/mgen.0.000436 PMC7660262 33001022

[21] Saati-Santamaría Z. 2023. Global map of specialized metabolites encoded in prokaryotic plasmids. Microbiol Spectr 11:e01523-23. doi:10.1128/spectrum.01523-23 37310275 PMC10434180

[22] diCenzo GC, Finan TM. 2017. The divided bacterial genome: structure, function, and evolution. Microbiol Mol Biol Rev 81:e00019-17. doi:10.1128/MMBR.00019-17 28794225 PMC5584315

[23] Medema MH, Trefzer A, Kovalchuk A, van den Berg M, Müller U, Heijne W, Wu L, Alam MT, Ronning CM, Nierman WC, Bovenberg RAL, Breitling R, Takano E. 2010. The sequence of a 1.8-mb bacterial linear plasmid reveals a rich evolutionary reservoir of secondary metabolic pathways. Genome Biol Evol 2:212–224. doi:10.1093/gbe/evq013 20624727 PMC2997539

[24] Harrison PW, Lower RPJ, Kim NKD, Young JPW. 2010. Introducing the bacterial “chromid”: not a chromosome, not a plasmid. Trends Microbiol 18:141–148. doi:10.1016/j.tim.2009.12.010 20080407

[25] Czarnecki J, Lamberioux M, Skovgaard O, Bignaud A, Taib N, Niault T, Bourhy P, Bos J, Krakowska E, Bartosik D, Koszul R, Marbouty M, Mazel D, Val M-E. 2025. Replication coordination marks the domestication of large extrachromosomal replicons in bacteria. bioRxiv. doi:10.1101/2025.03.15.643453 PMC13342612 42069678

[26] Uritskiy GV, DiRuggiero J, Taylor J. 2018. MetaWRAP-a flexible pipeline for genome-resolved metagenomic data analysis. Microbiome 6:158. doi:10.1186/s40168-018-0541-1 30219103 PMC6138922

[27] Chaumeil P-A, Mussig AJ, Hugenholtz P, Parks DH. 2020. GTDB-Tk: a toolkit to classify genomes with the Genome Taxonomy Database. Bioinformatics 36:1925–1927. doi:10.1093/bioinformatics/btz848 PMC7703759 31730192

[28] Chalita M, Kim YO, Park S, Oh H-S, Cho JH, Moon J, Baek N, Moon C, Lee K, Yang J, Nam GG, Jung Y, Na S-I, Bailey MJ, Chun J. 2024. EzBioCloud: a genome-driven database and platform for microbiome identification and discovery. Int J Syst Evol Microbiol 74:006421. doi:10.1099/ijsem.0.006421 38888585 PMC11261700

[29] Kolmogorov M, Yuan J, Lin Y, Pevzner PA. 2019. Assembly of long, error-prone reads using repeat graphs. Nat Biotechnol 37:540–546. doi:10.1038/s41587-019-0072-8 30936562

[30] Walker BJ, Abeel T, Shea T, Priest M, Abouelliel A, Sakthikumar S, Cuomo CA, Zeng Q, Wortman J, Young SK, Earl AM. 2014. Pilon: an integrated tool for comprehensive microbial variant detection and genome assembly improvement. PLoS One 9:e112963. doi:10.1371/journal.pone.0112963 25409509 PMC4237348

[31] Hunt M, Silva ND, Otto TD, Parkhill J, Keane JA, Harris SR. 2015. Circlator: automated circularization of genome assemblies using long sequencing reads. Genome Biol 16:294. doi:10.1186/s13059-015-0849-0 26714481 PMC4699355

[32] Olm MR, Brown CT, Brooks B, Banfield JF. 2017. dRep: a tool for fast and accurate genomic comparisons that enables improved genome recovery from metagenomes through de-replication. ISME J 11:2864–2868. doi:10.1038/ismej.2017.126 28742071 PMC5702732

[33] Capella-Gutiérrez S, Silla-Martínez JM, Gabaldón T. 2009. trimAl: a tool for automated alignment trimming in large-scale phylogenetic analyses. Bioinformatics 25:1972–1973. doi:10.1093/bioinformatics/btp348 19505945 PMC2712344

[34] Letunic I, Bork P. 2024. Interactive Tree of Life (iTOL) v6: recent updates to the phylogenetic tree display and annotation tool. Nucleic Acids Res 52:W78–W82. doi:10.1093/nar/gkae268 38613393 PMC11223838

[35] Balaban M, Moshiri N, Mai U, Jia X, Mirarab S. 2019. TreeCluster: clustering biological sequences using phylogenetic trees. PLoS One 14:e0221068. doi:10.1371/journal.pone.0221068 31437182 PMC6705769

[36] Blin K, Shaw S, Augustijn HE, Reitz ZL, Biermann F, Alanjary M, Fetter A, Terlouw BR, Metcalf WW, Helfrich EJN, van Wezel GP, Medema MH, Weber T. 2023. antiSMASH 7.0: new and improved predictions for detection, regulation, chemical structures and visualisation. Nucleic Acids Res 51:W46–W50. doi:10.1093/nar/gkad344 37140036 PMC10320115

[37] Paoli L, Ruscheweyh H-J, Forneris CC, Hubrich F, Kautsar S, Bhushan A, Lotti A, Clayssen Q, Salazar G, Milanese A, et al.. 2022. Biosynthetic potential of the global ocean microbiome. Nature 607:111–118. doi:10.1038/s41586-022-04862-3 35732736 PMC9259500

[38] Nayfach S, Roux S, Seshadri R, Udwary D, Varghese N, Schulz F, Wu D, Paez-Espino D, Chen I-M, Huntemann M, et al.. 2021. A genomic catalog of Earth’s microbiomes. Nat Biotechnol 39:499–509. doi:10.1038/s41587-020-0718-6 33169036 PMC8041624

[39] Kautsar SA, van der Hooft JJJ, de Ridder D, Medema MH. 2021. BiG-SLiCE: a highly scalable tool maps the diversity of 1.2 million biosynthetic gene clusters. Gigascience 10:giaa154. doi:10.1093/gigascience/giaa154 33438731 PMC7804863

[40] Gavriilidou A, Kautsar SA, Zaburannyi N, Krug D, Müller R, Medema MH, Ziemert N. 2022. Compendium of specialized metabolite biosynthetic diversity encoded in bacterial genomes. Nat Microbiol 7:726–735. doi:10.1038/s41564-022-01110-2 35505244

[41] Kautsar SA, Blin K, Shaw S, Weber T, Medema MH. 2021. BiG-FAM: the biosynthetic gene cluster families database. Nucleic Acids Res 49:D490–D497. doi:10.1093/nar/gkaa812 33010170 PMC7778980

[42] Zdouc MM, Blin K, Louwen NLL, Navarro J, Loureiro C, Bader CD, Bailey CB, Barra L, Booth TJ, Bozhüyük KAJ, et al.. 2025. MIBiG 4.0: advancing biosynthetic gene cluster curation through global collaboration. Nucleic Acids Res 53:D678–D690. doi:10.1093/nar/gkae1115 39657789 PMC11701617

[43] Krzywinski M, Schein J, Birol I, Connors J, Gascoyne R, Horsman D, Jones SJ, Marra MA. 2009. Circos: an information aesthetic for comparative genomics. Genome Res 19:1639–1645. doi:10.1101/gr.092759.109 19541911 PMC2752132

[44] Gourlé H, Karlsson-Lindsjö O, Hayer J, Bongcam-Rudloff E. 2019. Simulating Illumina metagenomic data with InSilicoSeq. Bioinformatics 35:521–522. doi:10.1093/bioinformatics/bty630 30016412 PMC6361232

[45] Li D, Liu C-M, Luo R, Sadakane K, Lam T-W. 2015. MEGAHIT: an ultra-fast single-node solution for large and complex metagenomics assembly via succinct de Bruijn graph. Bioinformatics 31:1674–1676. doi:10.1093/bioinformatics/btv033 25609793

[46] Wu Y-W, Simmons BA, Singer SW. 2016. MaxBin 2.0: an automated binning algorithm to recover genomes from multiple metagenomic datasets. Bioinformatics 32:605–607. doi:10.1093/bioinformatics/btv638 26515820

[47] Kang DD, Li F, Kirton E, Thomas A, Egan R, An H, Wang Z. 2019. MetaBAT 2: an adaptive binning algorithm for robust and efficient genome reconstruction from metagenome assemblies. PeerJ 7:e7359. doi:10.7717/peerj.7359 31388474 PMC6662567

[48] Alneberg J, Bjarnason BS, de Bruijn I, Schirmer M, Quick J, Ijaz UZ, Lahti L, Loman NJ, Andersson AF, Quince C. 2014. Binning metagenomic contigs by coverage and composition. Nat Methods 11:1144–1146. doi:10.1038/nmeth.3103 25218180

[49] Wang Z, You R, Han H, Liu W, Sun F, Zhu S. 2024. Effective binning of metagenomic contigs using contrastive multi-view representation learning. Nat Commun 15:585. doi:10.1038/s41467-023-44290-z 38233391 PMC10794208

[50] Parks DH, Imelfort M, Skennerton CT, Hugenholtz P, Tyson GW. 2015. CheckM: assessing the quality of microbial genomes recovered from isolates, single cells, and metagenomes. Genome Res 25:1043–1055. doi:10.1101/gr.186072.114 25977477 PMC4484387

[51] Gurevich A, Saveliev V, Vyahhi N, Tesler G. 2013. QUAST: quality assessment tool for genome assemblies. Bioinformatics 29:1072–1075. doi:10.1093/bioinformatics/btt086 23422339 PMC3624806

[52] Bowers RM, Kyrpides NC, Stepanauskas R, Harmon-Smith M, Doud D, Reddy TBK, Schulz F, Jarett J, Rivers AR, Eloe-Fadrosh EA, et al.. 2017. Minimum information about a single amplified genome (MISAG) and a metagenome-assembled genome (MIMAG) of bacteria and archaea. Nat Biotechnol 35:725–731. doi:10.1038/nbt.3893 28787424 PMC6436528

[53] Richter M, Rosselló-Móra R. 2009. Shifting the genomic gold standard for the prokaryotic species definition. Proc Natl Acad Sci USA 106:19126–19131. doi:10.1073/pnas.0906412106 19855009 PMC2776425

[54] Kim M, Oh H-S, Park S-C, Chun J. 2014. Towards a taxonomic coherence between average nucleotide identity and 16S rRNA gene sequence similarity for species demarcation of prokaryotes. Int J Syst Evol Microbiol 64:346–351. doi:10.1099/ijs.0.059774-0 24505072

[55] Zhu W, Klinman JP. 2020. Biogenesis of the peptide-derived redox cofactor pyrroloquinoline quinone. Curr Opin Chem Biol 59:93–103. doi:10.1016/j.cbpa.2020.05.001 32731194 PMC7736144

[56] Kadibalban AS, Landan G, Dagan T. 2024. The extent and characteristics of DNA transfer between plasmids and chromosomes. Curr Biol 34:3189–3200. doi:10.1016/j.cub.2024.06.030 38964320

[57] Yabe S, Wang C-M, Zheng Y, Sakai Y, Abe K, Yokota A. 2020. Formation of sporangiospores in Dictyobacter aurantiacus (class Ktedonobacteria in phylum Chloroflexi). J Gen Appl Microbiol 65:316–319. doi:10.2323/jgam.2019.01.001 31118349

[58] Ji M, Greening C, Vanwonterghem I, Carere CR, Bay SK, Steen JA, Montgomery K, Lines T, Beardall J, van Dorst J, Snape I, Stott MB, Hugenholtz P, Ferrari BC. 2017. Atmospheric trace gases support primary production in Antarctic desert surface soil. Nature 552:400–403. doi:10.1038/nature25014 29211716

[59] Kautsar SA, Blin K, Shaw S, Navarro-Muñoz JC, Terlouw BR, van der Hooft JJJ, van Santen JA, Tracanna V, Suarez Duran HG, Pascal Andreu V, Selem-Mojica N, Alanjary M, Robinson SL, Lund G, Epstein SC, Sisto AC, Charkoudian LK, Collemare J, Linington RG, Weber T, Medema MH. 2019. MIBiG 2.0: a repository for biosynthetic gene clusters of known function. Nucleic Acids Res 48:D454–D458. doi:10.1093/nar/gkz882 PMC7145714 31612915

[60] Westhoff S, Kloosterman AM, van Hoesel SFA, van Wezel GP, Rozen DE. 2021. Competition sensing changes antibiotic production in Streptomyces mBio 12:e02729-20. doi:10.1128/mBio.02729-20 PMC7885098 33563841

[61] Flärdh K, Buttner MJ. 2009. Streptomyces morphogenetics: dissecting differentiation in a filamentous bacterium. Nat Rev Microbiol 7:36–49. doi:10.1038/nrmicro1968 19079351

[62] Chase AB, Sweeney D, Muskat MN, Guillén-Matus DG, Jensen PR. 2021. Vertical inheritance facilitates interspecies diversification in biosynthetic gene clusters and specialized metabolites. mBio 12:e02700-21. doi:10.1128/mBio.02700-21 34809466 PMC8609351

[63] Ludueña LM, Anzuay MS, Angelini JG, Barros G, Luna MF, Monge M del P, Fabra A, Taurian T. 2017. Role of bacterial pyrroloquinoline quinone in phosphate solubilizing ability and in plant growth promotion on strain Serratia sp. S119. Symbiosis 72:31–43. doi:10.1007/s13199-016-0434-7

[64] diCenzo GC, Mengoni A, Perrin E. 2019. Chromids aid genome expansion and functional diversification in the family Burkholderiaceae. Mol Biol Evol 36:562–574. doi:10.1093/molbev/msy248 30608550

[65] Liu H, Sun J, Si J, Liao Y, Bai J, Li X, Wang L, Cai K, Ni W, Zhou P, Hu S. 2025. Unexplored diversity and potential functions of extra-chromosomal elements. mSystems 10:e00175-25. doi:10.1128/msystems.00175-25 40827884 PMC12456017

[66] Chevrette MG, Gutiérrez-García K, Selem-Mojica N, Aguilar-Martínez C, Yañez-Olvera A, Ramos-Aboites HE, Hoskisson PA, Barona-Gómez F. 2020. Evolutionary dynamics of natural product biosynthesis in bacteria. Nat Prod Rep 37:566–599. doi:10.1039/c9np00048h 31822877

[67] Jiang W, Pan J, Lin T, Wang Y, Wang Y, Zhang R, Zhou X, Zhang Y. 2025. Mutational features of chromids and chromosomes in Pseudoalteromonas provide new insights into the evolution of secondary replicons. Microbiol Spectr 13:e02127-24. doi:10.1128/spectrum.02127-24 40130865 PMC12053903

[68] Simon G, Casalot L, Valette C, Burot C, Rontani JF, Bonin P. 2025. Do carotenoids protect phytodetritus-associated bacteria from oxidative stress? Environ Sci Pollut Res 32:11167–11178. doi:10.1007/s11356-025-36080-5 PMC12014824 40198437

