# Supplementary material for: Chromid-like secondary replicons as predicted key sites of biosynthetic gene clusters in Ktedonobacteria
Source: mSystems. 2026 May 29;11(6):e00197-26. doi: 10.1128/msystems.00197-26 (PMC13289182; doi:10.1128/msystems.00197-26)
Supplement: Supplemental Figures — Figures S1-S7. [file msystems.00197-26-s0001.pdf]

Supplementary information for

Chromid-like secondary replicons as key sites of predicted biosynthetic gene clusters in *Ktedonobacteria*

Shuhei Yabe, Yu Zheng, Shunji Takahashi, Chongyang Yang, Yui Nose, Shinichi Yamazaki, Nao Okuma, Mazytha Kinanti Rachmania, Fitria Ningsih, Wellyzar Sjamsuridzal, Mayuko Sato, Kiminori Toyooka, and Yasunori Ichihashi

Shuhei Yabe
shuhei.yabe@riken.jp

Yasunori Ichihashi
yasunori.ichihashi@riken.jp

This PDF includes:

Supplementary Figure S1 to Supplementary Figure S7

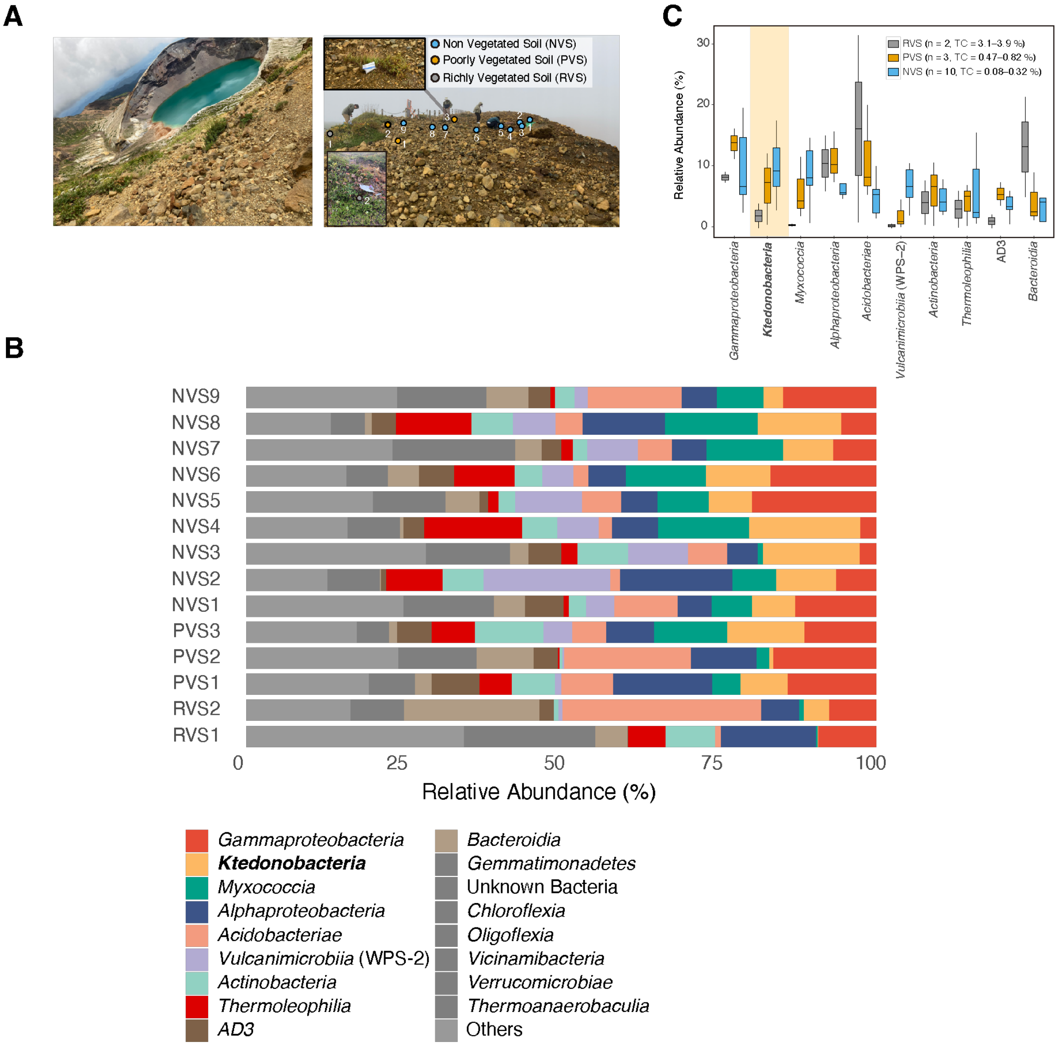

Supplementary Fig. S1 Sampling design and class-level bacterial composition in soils from Zao Volcano.

(A) Sampling sites. Left: panoramic view of the crater lake Okama at Mt. Zao, Japan. Right: close-up of the sampling area, showing non-vegetated soils (NVS), poorly vegetated soils (PVS), and richly vegetated soils (RVS). Sampling points are color-coded according to vegetation cover and associated total organic carbon (TOC) content: RVS (n = 2, TOC = 2.04%), PVS (n = 3, TOC = 0.46–0.99%), and NVS (n = 10, TOC = 0.25–0.41%).

(B) Class-level bacterial community composition in individual soil samples. Stacked bar plots show the relative abundance of bacterial classes across NVS, PVS, and RVS. Taxa with >5% relative abundance in at least one sample are shown individually; all others are grouped as "Others." Taxonomic assignment is based on 16S rRNA gene sequences.

(C) Relative abundances of the ten most abundant bacterial classes in NVS and PVS soils, based on 16S rRNA gene (V4 region) amplicon sequencing. Boxes indicate interquartile ranges with medians.

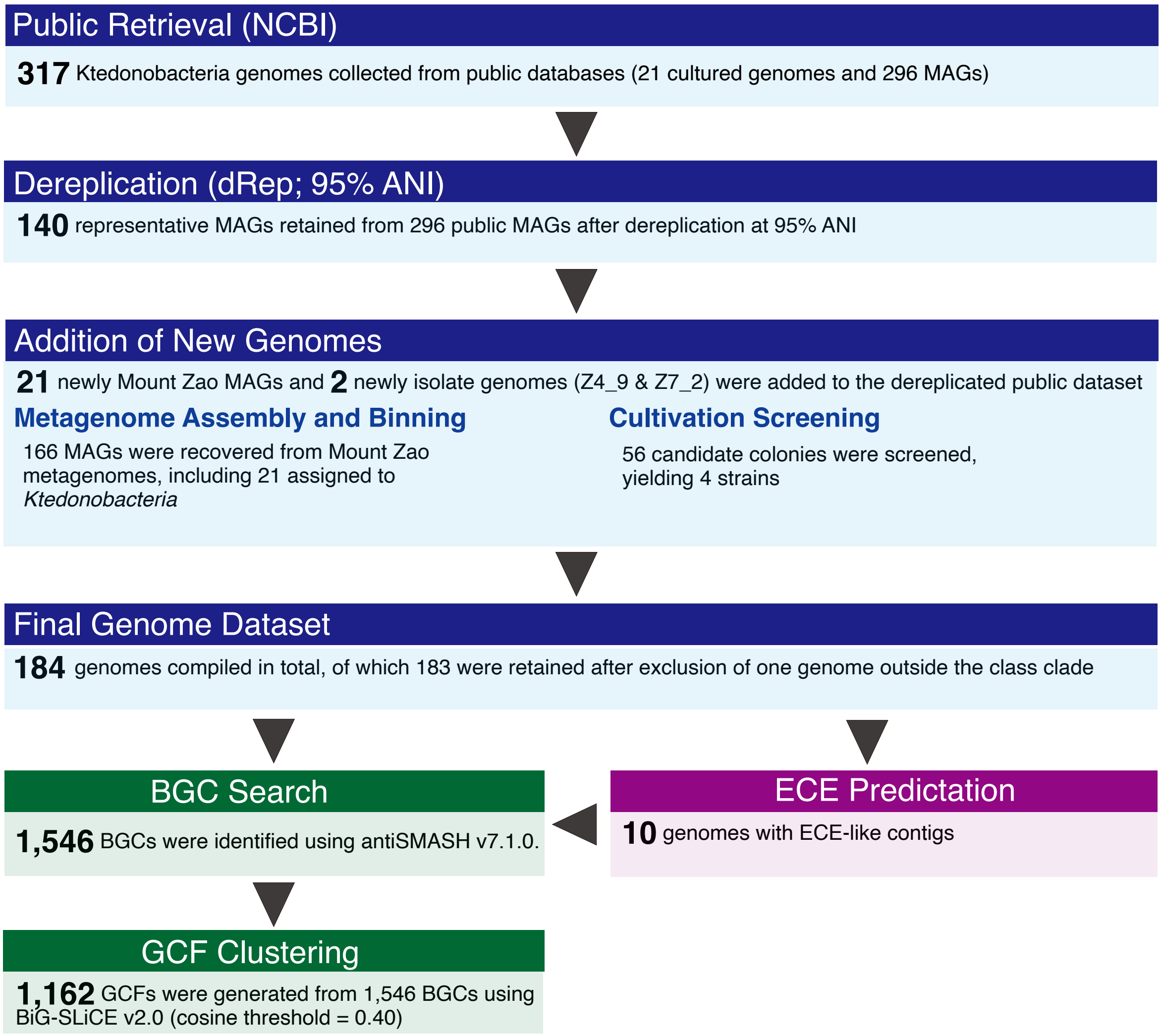

Supplementary Fig. S2 Workflow for genome dataset construction and downstream analyses

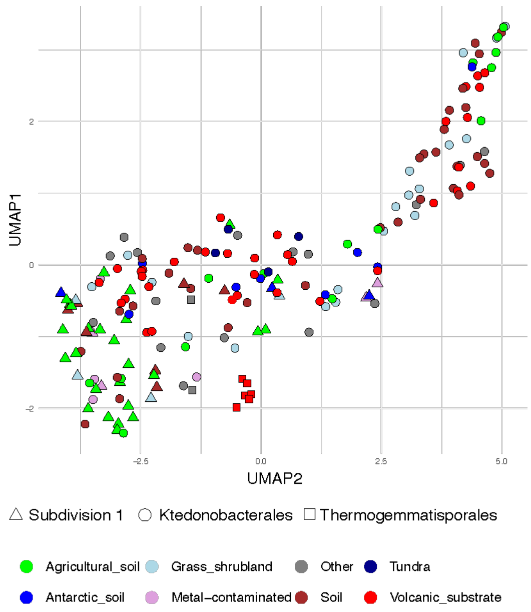

Supplementary Fig. S3 UMAP projection of BGC subclass profiles across Ktedonobacteria genomes. Each point represents a genome projected based on its BGC subclass profile derived from BiG-SLiCE v2.0 classification. This analysis provides an overview of the biosynthetic profile structure underlying the lineage- and habitat-associated patterns shown in Fig. 3A and B. Shapes indicate major phylogenetic groups, and colors indicate broad ecosystem categories derived from BioSample metadata. UMAP1 and UMAP2 represent reduced dimensions of biosynthetic profile dissimilarity.

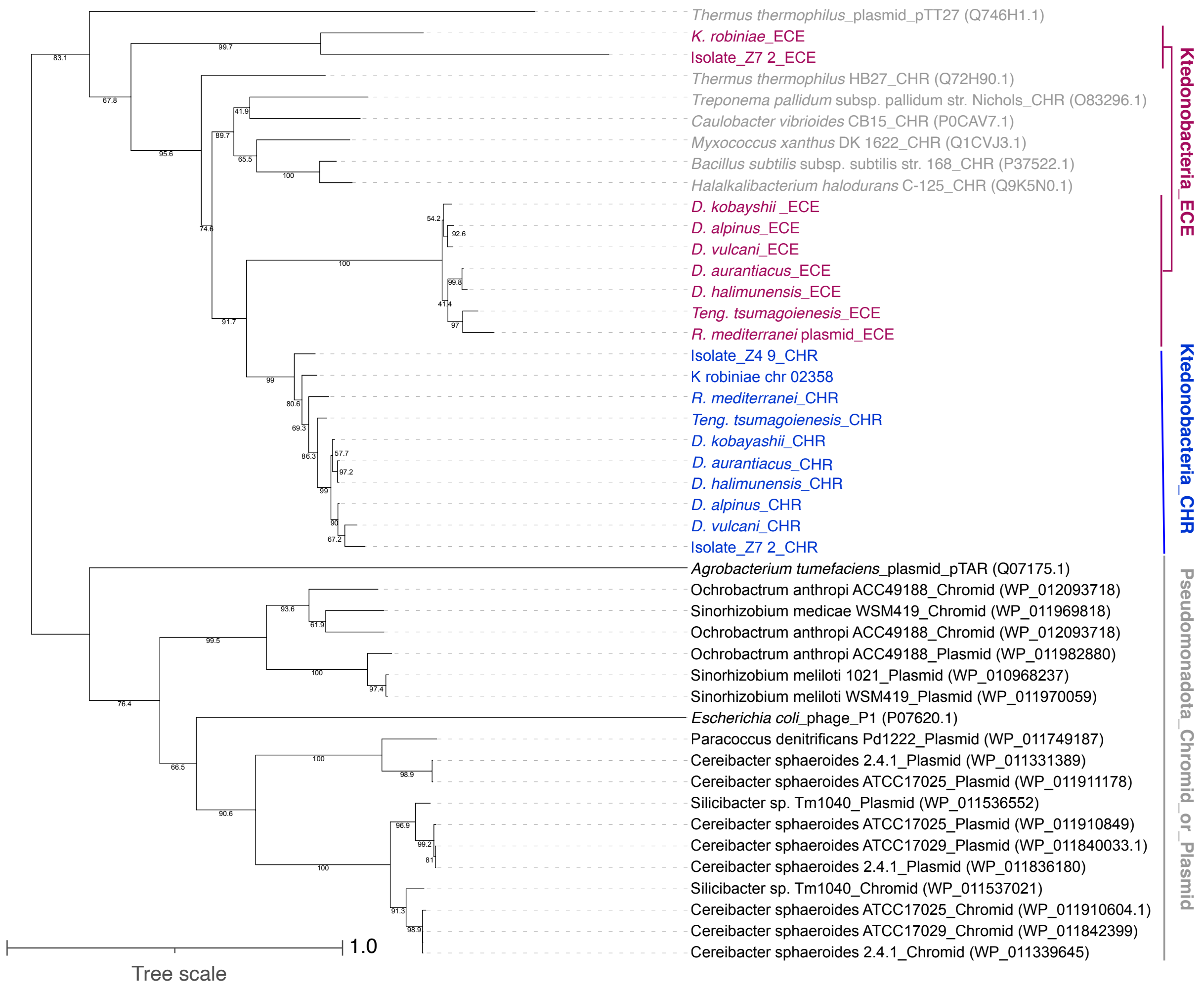

Supplementary Fig. S4 Maximum-likelihood phylogeny of ParA/RepA homologs from representative CHR- and ECE-like contigs.

Protein sequences were aligned with MUSCLE v3.8.425, trimmed with trimAl (gap threshold = 0.5, similarity threshold = 60), and analyzed in IQ-TREE v2.3.6 under the Q.pfam+I+G4 substitution model. Branch support values are shown as SH-aLRT (left) and ultrafast bootstrap (right) percentages (both with 1,000 replicates).

Ktedonobacteria sequences are highlighted in red (ECE-like contig-derived) and blue (CHR-like contigs), while other representative taxa are shown in black.

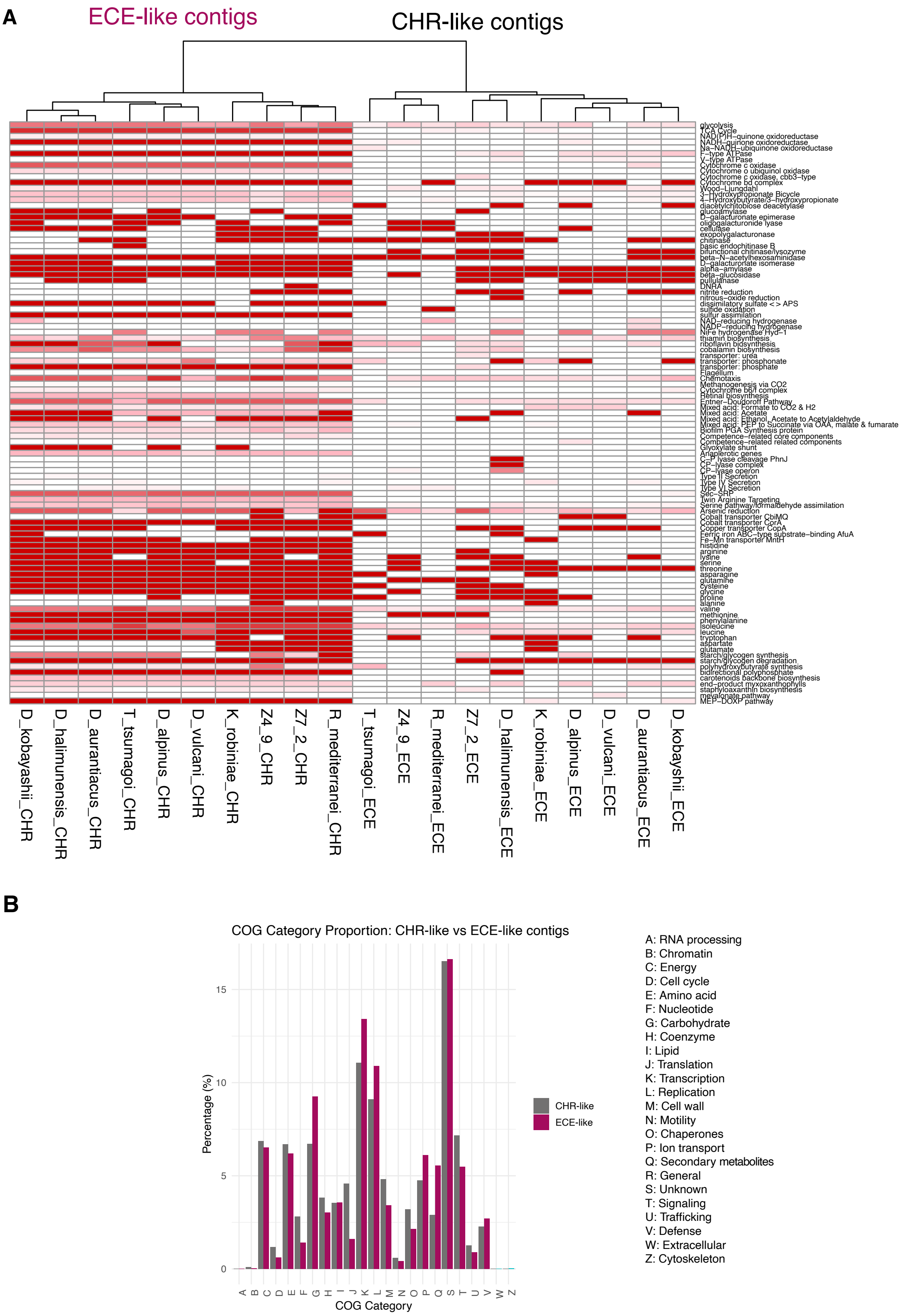

Supplementary Fig. S5 Functional partitioning between CHR-like and ECE-like contigs in *Ktedonobacteria*.

(A) Heatmap of KEGG functional categories (rows) across contigs (columns) from the isolates

shown. Columns are hierarchically clustered and grouped by replicon type: ECE-like and CHR-like contigs. Cell color indicates the normalized representation (0–1) of genes assigned to each category within a contig. Functional annotation used eggNOG-mapper v2.1.12 (eggNOG v5.0.2), and KEGG terms were parsed from its output.

(B) Relative proportions of COG one-letter functional categories on CHR-like (grey) and ECE-like contigs (magenta) computed from the same annotations; category labels (A–Z) are listed at right.

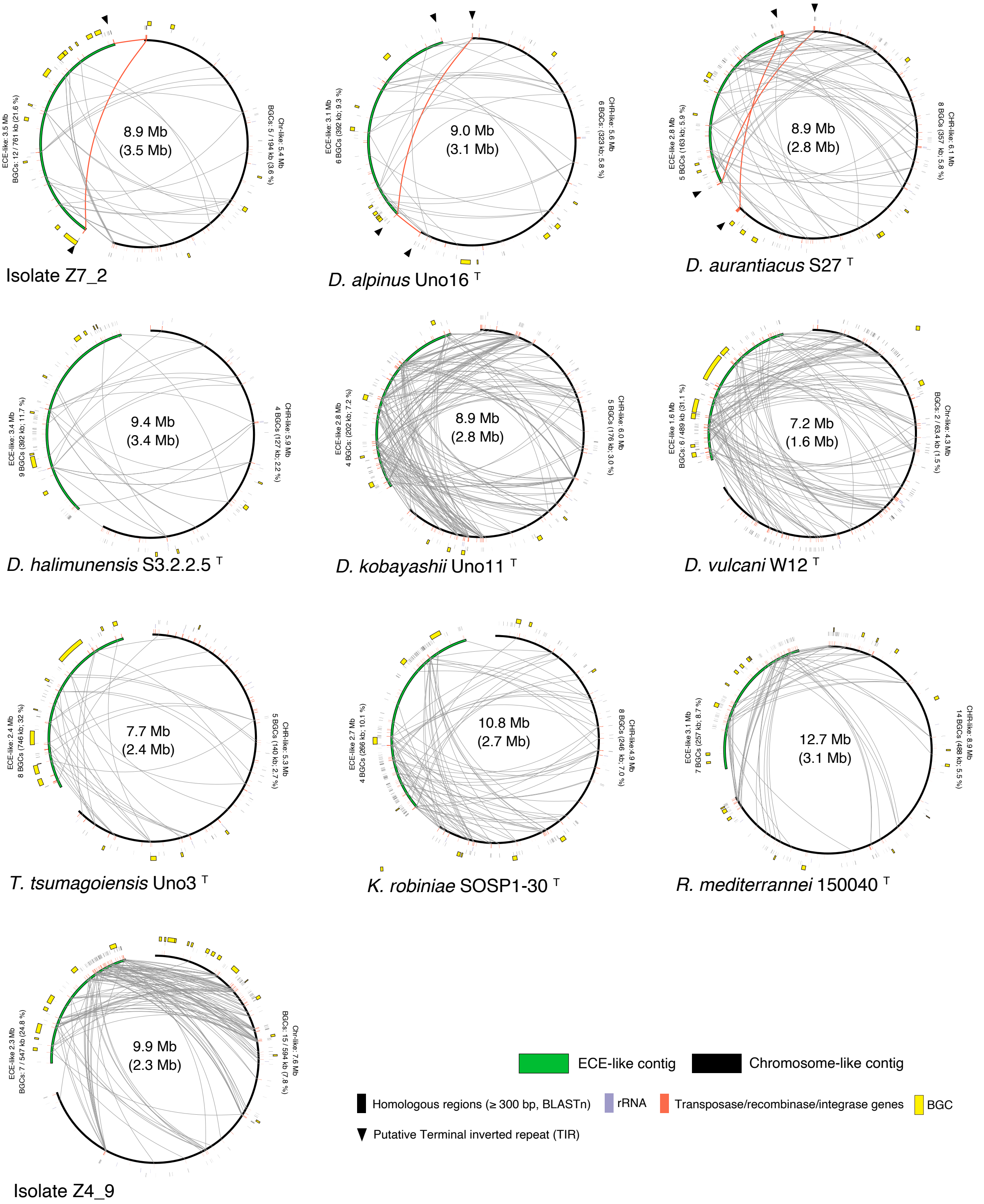

Supplementary Fig. S6 Shared homologous regions and distribution of BGCs and mobility genes on CHR- and ECE-like contigs.

Circular maps are shown for ten strains. The CHR- and ECE-like contigs are indicated in black and green, respectively. Grey ribbons connect pairwise homologous regions detected by BLASTn (identity ≥ 98%, length ≥ 300 bp). Orange ribbons indicate long, high-identity

homologous segments (length ≥ 5 kb, identity ≥ 99%). Black triangles denote putative TIRs. Gene features are overlaid as follows: yellow boxes, BGCs (predicted with antiSMASH v7.1.0); red ticks, mobility-associated genes (integrases, recombinases, and transposases); purple ticks, rRNA operons. The centre of each panel shows the total length of the CHR–ECE pair, with the ECE-like contig length in parentheses. For each replicon, ring-side labels report its length and BGC burden in the format “8 BGCs (746 kb; 32%)”. Each percentage indicates the fraction of the corresponding replicon (CHR- or ECE-like contig) covered by BGCs.

TIR, terminal inverted repeat; ECE, extrachromosomal element; BGC, biosynthetic gene cluster; CHR, chromosome.

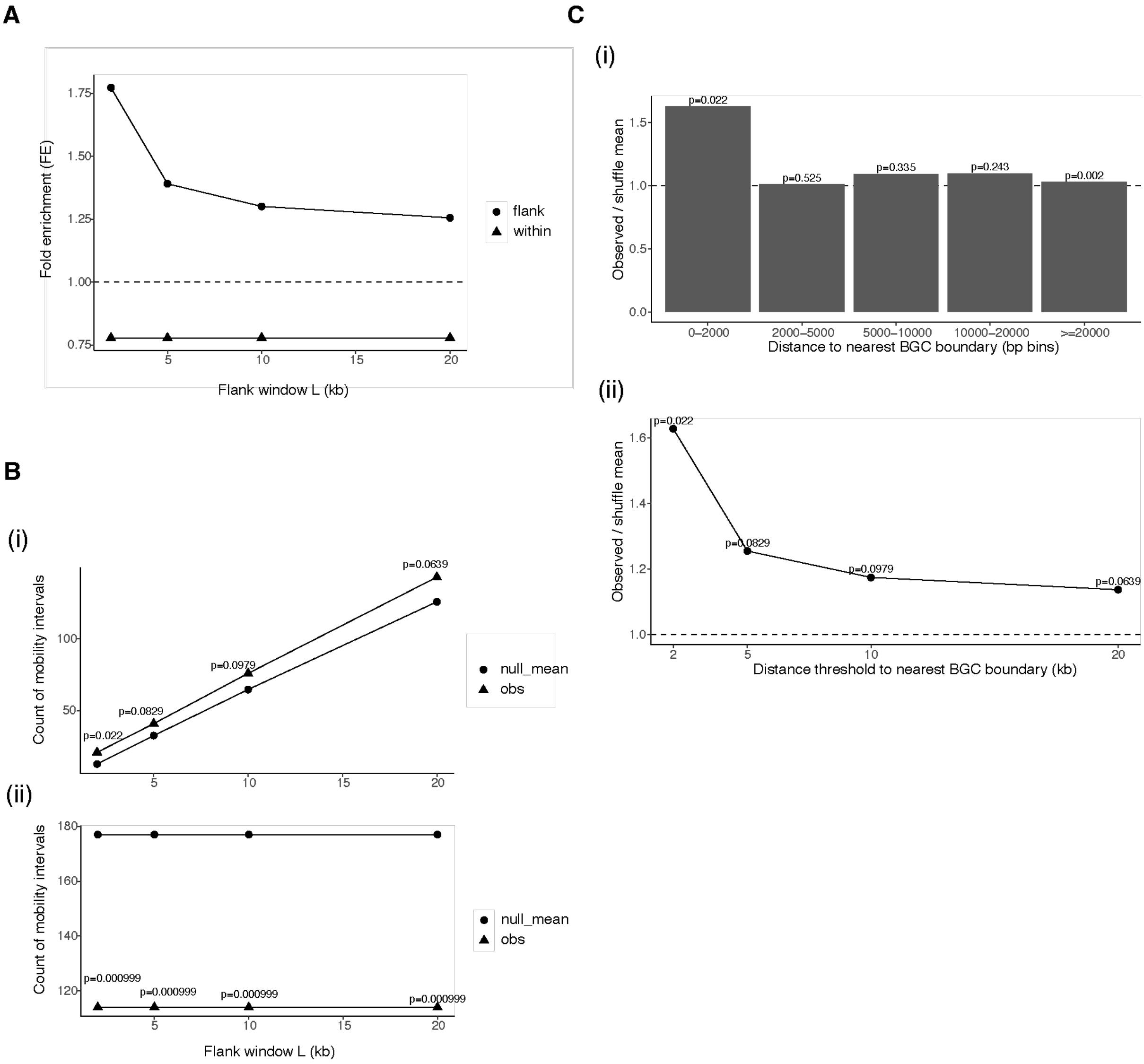

Supplementary Fig. S7 Spatial association between mobility loci and biosynthetic gene clusters

Spatial relationships between mobility loci and biosynthetic gene clusters (BGCs) assessed using length-corrected enrichment analyses and contig-restricted permutation tests.

(A) Fold enrichment (FE) of mobility loci within BGC bodies (within; triangles) and in flanking regions surrounding BGCs (flank; circles) across different flank window sizes (*L* = 2, 5, 10, and 20 kb).

Fold enrichment was calculated as the ratio of the observed fraction of mobility loci in a given region to the fraction expected under a random distribution proportional to region length. The dashed horizontal line indicates the expectation under random distribution (FE = 1).

(B) Robustness of the enrichment patterns evaluated by contig-restricted shuffling (1000 permutations), in which mobility intervals were randomly repositioned within each replicon while preserving interval lengths.

(i) Observed counts of mobility loci in BGC flanking regions (triangles) compared with the

mean expected counts from shuffling (circles).
(ii) Observed counts of mobility loci overlapping BGC bodies (triangles) compared with shuffle-based expectations (circles).
Empirical one-sided permutation *P* values are shown for each window size.
(C) Distance-based analysis of mobility loci relative to BGC boundaries. Mobility loci overlapping BGC bodies were excluded from this analysis.
(i) Enrichment in discrete distance bins, shown as the ratio of observed counts to the shuffle mean for mobility loci located at increasing distances from the nearest BGC boundary.
(ii) Cumulative enrichment of mobility loci located within the indicated distance thresholds from the nearest BGC boundary.
The dashed horizontal lines in panels (b) and (c) indicate expectations under the shuffle-based null model (observed/shuffle mean = 1).
Together, these analyses show that mobility loci are depleted within BGC bodies but preferentially accumulate in the immediate vicinity of BGC boundaries, with the strongest enrichment observed within approximately 2 kb of BGC border.
